# Archaeal nitrification is constrained by copper complexation with organic matter in municipal wastewater treatment plants

**DOI:** 10.1038/s41396-019-0538-1

**Published:** 2019-10-17

**Authors:** Joo-Han Gwak, Man-Young Jung, Heeji Hong, Jong-Geol Kim, Zhe-Xue Quan, John R. Reinfelder, Emilie Spasov, Josh D. Neufeld, Michael Wagner, Sung-Keun Rhee

**Affiliations:** 1 Department of Microbiology, Chungbuk National University, 1 Chungdae-ro, Seowon-Gu, Cheongju, 28644 South Korea; 2 Division of Microbial Ecology, Department of Microbiology and Ecosystem Science, Centre for Microbiology and Environmental Systems Science, University of Vienna, Althanstrasse 14, A-1090 Vienna, Austria; 3 Ministry of Education Key Laboratory for Biodiversity Science and Ecological Engineering, Institute of Biodiversity Science, School of Life Sciences, Fudan University, Shanghai, China; 4 Department of Environmental Sciences, Rutgers University, New Brunswick, NJ 08901 USA; 5 Department of Biology, University of Waterloo, Waterloo, ON N2L 3G1 Canada; 6 Center for Microbial Communities, Department of Chemistry and Bioscience, Aalborg University, Fredrik Bajers Vej 7H, 9220 Aalborg, Denmark

**Keywords:** Microbial ecology, Ecosystem ecology, Archaeal physiology

## Abstract

Consistent with the observation that ammonia-oxidizing bacteria (AOB) outnumber ammonia-oxidizing archaea (AOA) in many eutrophic ecosystems globally, AOB typically dominate activated sludge aeration basins from municipal wastewater treatment plants (WWTPs). In this study, we demonstrate that the growth of AOA strains inoculated into sterile-filtered wastewater was inhibited significantly, in contrast to uninhibited growth of a reference AOB strain. In order to identify possible mechanisms underlying AOA-specific inhibition, we show that complex mixtures of organic compounds, such as yeast extract, were highly inhibitory to all AOA strains but not to the AOB strain. By testing individual organic compounds, we reveal strong inhibitory effects of organic compounds with high metal complexation potentials implying that the inhibitory mechanism for AOA can be explained by the reduced bioavailability of an essential metal. Our results further demonstrate that the inhibitory effect on AOA can be alleviated by copper supplementation, which we observed for pure AOA cultures in a defined medium and for AOA inoculated into nitrifying sludge. Our study offers a novel mechanistic explanation for the relatively low abundance of AOA in most WWTPs and provides a basis for modulating the composition of nitrifying communities in both engineered systems and naturally occurring environments.

## Introduction

Nitrification, the aerobic oxidation of ammonia via nitrite to nitrate, is a key process of the global biogeochemical nitrogen cycle. In industrial and municipal wastewater treatment plants (WWTPs), nitrification (in combination with denitrification or anammox) is critical for nitrogen removal and thus is essential for reducing ammonia toxicity to aquatic life and eutrophication in receiving water bodies. Ammonia conversion to nitrite, the first and rate-limiting step of nitrification is mediated by ammonia-oxidizing bacteria (AOB) and ammonia-oxidizing archaea (AOA), but can also be performed by the recently discovered complete ammonia-oxidizing bacteria (comammox), which, in contrast to AOA and AOB, further oxidize the formed nitrite to nitrate [1, 2].

AOA outnumber their bacterial counterparts in many oligotrophic terrestrial and marine habitats [3–5] often by several orders of magnitude, while AOB populations typically dominate under eutrophic conditions [6–8]. Consistently, AOB control ammonia oxidation in almost all municipal and many industrial WWTPs [9–11]. However, interestingly in certain industrial wastewater treatment systems, in some municipal activated sludge systems with low ammonia effluent values and in some municipal plants employing alternatives to activated sludge-based systems AOA dominance has been described [12–15], but the actual factors causing this unusual composition of the nitrifying community are unknown. AOA/AOB ratios might be depended on the treatment process and operating conditions [13, 14, 16]. In addition, substrate affinity, mixotrophy, metal toxicity, temperature, and pH may contribute to niche differentiation between AOA and AOB [17–20]. Detailed knowledge of factors that differentially influence the growth of AOA and AOB is important for predicting and controlling nitrifying community composition and WWTP performance.

Microorganisms in WWTPs are exposed to high concentrations of both solid and dissolved organic matter. Although the composition and concentration of organic matter may be a key factor that governs the relative abundance and activity of AOA and AOB in environments [20], the relationship of AOA to organic carbon is poorly characterized, and controversially discussed. For example, it is still not clear whether some ammonia oxidizers may be capable of mixotrophy. Organic carbon assimilation by AOA could result in a higher growth rate, increased cell biomass yields, and a competitive advantage over a strictly autotrophic lifestyle. In support of a mixotrophic metabolism for several AOA representatives, Schauss et al. [21] showed that AOA growth was stimulated by the addition of organic matter. In addition, Mußman et al. [12] reported lack of CO_2_ fixation by a clade of AOA group I.1b related to “*Candidatus* Nitrosocosmicus” that was abundant in a refinery nitrifying sludge. Similarly, “*Ca*. Nitrosocosmicus exaquare” assimilated bicarbonate in an enrichment culture, but bicarbonate assimilation by AOA was not observed in the tertiary treatment system biofilm from which it was obtained [22]. Furthermore, Jia and Conrad [23] showed that AOB were primarily responsible for soil nitrification, whereas AOA growth proceeded independently from total nitrification, suggesting assimilation of organic carbon. On the other hand, inhibition of growth of AOA strains by organic compounds is also commonly observed (for a literature summary see Table S1). Furthermore, stimulatory effects of α-ketoic acids (i.e., pyruvate and α-ketoglutarate) on archaeal ammonia oxidation were recently demonstrated as being linked to scavenging of reactive oxygen species (especially H_2_O_2_) rather than mixotrophy [24].

In the current study, we investigated inhibition profiles of representative AOA and AOB strains by various organic compounds and sterile-filtered wastewater. AOA were clearly more sensitive to inhibition by organic compounds than the tested AOB representative. Furthermore, we revealed that the observed inhibition of AOA growth was caused by copper complexation by the organic compounds, suggesting that differences in copper requirements and acquisition mechanisms between AOA and AOB likely explain their differential sensitivities to organic compounds. In addition, we show that copper amendments dramatically reduce inhibition of AOA by organic compounds and even allow for growth of an AOA strain in municipal nitrifying activated sludge. Our results demonstrate that limited copper bioavailability is a key factor constraining the activity of archaeal ammonia oxidation in municipal WWTPs and likely many other organic-rich ecosystems and that copper bioavailability is an important factor contributing to niche differentiation between AOA and AOB.

## Materials and methods

### Cultivation of AOA and AOB

To reduce trace metal contamination, polycarbonate bottles (Nalgene) and polypropylene or polystyrene bottles (Falcon) were used to cultivate AOA and AOB strains (Table S2) after soaking the bottles for 24 h in 10% v/v HCl (Trace Metal Grade; OCI, Korea) and rinsing them three times with Milli-Q water. Ultrapure water (Ultra Trace Elemental Analysis Grade; Fisher Scientific) was used for preparing all media and stock solutions. Basal mineral salts solution (see details in Table S3) and all additional components were filter sterilized using 0.1 µm pore-size polyethersulfone (PES) syringe filters (Sartorius, Germany) that were prewashed with 1 M HCl and rinsed with Milli-Q water. To prepare artificial freshwater medium (AFM) [25], after filter sterilization of the basal salts solution, NH_4_Cl (1 mM), NaHCO_3_ (2 mM), HEPES (pH 7.4; 3 mM), 0.1 mL (0.1×) vitamin solution, and 0.1 mL (0.1×) trace metals solution (TMS; see details in Table S3) per liter were added unless stated otherwise. Cultures were incubated under oxic conditions with ambient air and without shaking in the dark at 25 °C (*Nitrosachaeum koreense* and *Nitrosomonas europaea*), 30 °C (*Ca*. Nitrosotenuis chungbukensis and *Ca*. Nitrosocosmicus oleophilus), or 42 °C (*Nitrososphaera viennensis*) (Table S2). Sodium pyruvate (0.1 mM) was used as a hydrogen peroxide (H_2_O_2_) scavenger [24] for cultivation of *N. koreense*, *Ca*. N. chungbukensis, and *N. viennensis* in all experiments. The pH of the medium was adjusted to 7.0–7.5 by 1 M NaOH or 1 M HCl and remained constant throughout oxidation of 1 mM ammonia by AOA and AOB strains. The growth of AOA and AOB was determined by measuring ammonia and nitrite concentrations as previously described [25]. For microscopic counting of total cells, SYBR Gold staining was used after the cells were filtered (0.2 µm polycarbonate GTTP membranes; Merck Millipore, Germany) according to a previously published protocol [25].

### Inhibitory effects of organic compounds

To investigate the effect of yeast extract on the growth of AOA and AOB, yeast extract (0, 5, 10, and 50 mg L^−1^) was added to AFM with 1× TMS before inoculation with ammonia oxidizers. In addition, media amendments with other complex organic compound mixtures (humic acid, tryptone, and peptone at 50 mg L^−1^) as well as selected common organic acids and amino acids (acetate, gluconate, salicylate, citrate, l-aspartate, l-cysteine, l-histidine, l-arginine, l-glutamate, l-lysine, and l-valine at 0.5 mM, corresponding to 30–97 mg L^−1^) were performed for testing their inhibitory effect on AOA and AOB growth in the AFM. Stock solutions of the organic compounds were prepared by dissolving them in ultrapure water (Ultra Trace Elemental Analysis Grade; Fisher) and filter sterilizing the solutions using 0.1 µm pore-size PES syringe filters that were prewashed with 1 M HCl and rinsed with Milli-Q water. One percent (v/v) cultures of AOA and AOB strains (Table S2) at late log phase were harvested, washed, and then inoculated into the media, unless otherwise specified. Throughout this study, nitrite concentration instead of cell counts was used to calculate specific growth rates because close correlations of cell growth and nitrite production during ammonia oxidation were consistently observed for the tested strains (*R*^2^ = 0.99; unpublished data) as previously published for other AOA strains [25, 26]. The specific growth rate was calculated by determining the slope according to the equation μ = (ln*N*_1 _− ln*N*_0_)/(*t*_1_ − *t*_0_), where (ln*N*_1_ − ln*N*_0_) is the change in the natural log of nitrite concentration and (*t*_1_ − *t*_0_) is the change in time.

Restoration of growth of AOA inhibited by organic compounds was investigated by augmentation of TMS to the AFM amended with inhibitory organic compounds. Complex organic compound mixtures (yeast extract, tryptone, peptone; 50 mg L^−1^) or histidine (0.5 mM) were added to the AFM to inhibit growth of AOA. Various concentrations of TMS (4×, 8×, 10×, 20×, and 40× for histidine; 1× and 2× for complex mixtures of organic compounds) were added to the AFM. Further, restoration AOA strain growth that was constrained by yeast extract and histidine was investigated by augmentation of individual trace metals of the TMS (Table S3) to the AFM. Various concentrations of individual metals (5×, 10×, and 20×concentration of that in the TMS for yeast extract; 20×, 80×, and 320× concentration of that in the TMS for histidine) were added to the AFM. Because of additional inorganic iron(III) precipitates in the AFM, iron(III) citrate was used. All other metals were added as salts that were also used for preparation of the TMS (Table S3).

### Growth experiment in wastewaters of aerobic reactors in municipal WWTPs

Mixed liquor samples of aerobic nitrifying reactors of municipal WWTPs from three Korean metropolitan cities (Daejeon, DJ; Cheongju, CJ; Bucheon, BC) (see Table 1) were collected in August–December 2016. In addition, a mixed liquor sample from the nitrifying reactor of Refinery D [12] and of the nitrifying rotating biological contactors (RBCs) that represent the tertiary treatment system of a municipal WWTP [14, 22] (RBC 1 and RBC 8; Guelph, Canada) were collected in October 2017. Relatively high AOA abundances were previously reported in the refinery plant reactor and the RBC systems [12, 14, 22]. Details regarding these plants, including operation conditions and origin of the wastewaters, are described in Table 1. All collected mixed liquor samples of CJ, DJ, and BC were transported to the laboratory at 4 °C. To obtain sterile-filtered wastewater samples, the liquid phase was separated from suspended solids by centrifugation at 4000 × *g* for 30 min, then filter sterilized using 0.2 µm pore-size PES membranes (Sartorius, Germany) that were prewashed with 1 M HCl and rinsed with Milli-Q water. For the Refinery D, RBC 1, and RBC 8 samples, filter-sterilized wastewater samples were transported to the laboratory in Korea at 4 °C.

**Table 1:** Operational data of wastewater treatment plants investigated in this study

	Daejeon (DJ)	Cheongju (CJ)	Bucheon (BC)	Oil refinery D	RBC 1^a^	RBC 8^a^
Location	Daejon, Korea	Cheongju, Korea	Bucheon, Korea	UK	Guelph, Canada	Guelph, Canada
Origin of wastewater	Domestic	Domestic	Domestic	Oil refinery	Domestic	Domestic
Treatment process^b^	A^2^/O	CNR	DeNiPho	Conventional	RBC	RBC
Flow rate (m^3^ day)	565,000	222,000	389,000	3900	54,000	54,000
Hydraulic retention time (h)	17.5	4–8	18	–	0.89	0.89
MLSS (mg L^−1^)	3500–3900	2,700–2,800	2600–2700	–	N.A.	N.A.
Influent COD (BOD)^c^ (mg L^−1^)	103.5 (195)	82 (152)	65 (122)	951 (344)	N.A.	N.A.
Influent NH_3_-N (mg L^−1^)	32.1	39.7	27.4	12	0.52	0.06
Effluent NH_3_-N (mg L^−1^)	0.38	7.7	0.79	0.3	0.52	0.06
Effluent NO_3_^−^-N + NO_2_^−^-N (mg L^−1^)	29.9	7.4	37.5	17.1	25.5	25.2
pH	6.7	6.6	6.5	7.0	7.2	7.5
DOC (mg L^−1^)	13.2	14.7	14.0	24.2	14.9	17.2
DOC after UV treatment (mg L^−1^)	1.8	3.4	3.0	N.D.	N.D.	N.D.
Cu (μg L^−1^)	1.6	0.8	1.5	1.7	2.8	3.8
Fe (μg L^−1^)	16.1	9.1	7.5	24.8	41.2	49.1
Ni (μg L^−1^)	8.4	3.5	48.8	4.7	6.6	6.8
Zn (μg L^−1^)	19.0	13.4	29.5	3.7	41.7	45.2
Co (μg L^−1^)	1.7	0.4	1.1	4.0	1.6	1.5
Mn (μg L^−1^)	76.8	77.3	93.8	24.1	10.8	6.3

pH, MLSS, DOC, and trace metals were determined for samples of the mixed liquor from aerobic reactors where nitrification occurs

^a^The rotating biological contactors (RBCs) represent the tertiary treatment system of the municipal wastewater treatment plant in Guelph, Ontario. The water flows through eight RBCs in series with four trains of RBCs treating wastewater along four flow paths. Thus, representative RBC 1 and RBC 8 data are presented as generally as possible, given that multiple trains and time point data are available. Data are summarized from lab analyses of the authors, annual reports from the WWTP, or from Sauder et al. [22]

^b^*A*^2^/*O* anaerobic-anoxic-oxic wastewater process, *CNR* cilium nutrient removal wastewater process, *DeNiPho* DeNiPho wastewater process, *RBC* rotating biological contactor wastewater process

^c^*COD* chemical oxygen demand, *BOD* biochemical oxygen demand

*N.A.* not applicable, *N.D.* no data

To investigate growth of AOA and AOB strains in filtered wastewater of the nitrifying aerobic reactors from three municipal WWTPs (DJ, CJ, and BC) and from the WWTPs with high AOA abundances (Refinery D, RBC 1, and RBC 8), AOA and AOB cells (Table S2) were washed before inoculation to avoid transferring trace metals from the culture medium. Cells at late log phase were harvested by centrifugation at 1000 × *g* for 20 min using Amicon Ultra-15 centrifugal filter units (Ultracel-100K; Merck Millipore, Germany) and resuspended in trace metal-free AFM. After washing two more times, resuspended cells (1%, v/v) were inoculated into filtered wastewater. Before inoculation, the filtered wastewater was supplemented with NH_4_Cl (1 mM) and HEPES (pH 7.4; 1 mM). After adding supplements, the pH range of the filtered wastewater from the different plants was always between 7.2 and 7.4. To assess copper requirement for growth of AOA and AOB in WWTPs (CJ, DJ, and BC), various amounts of copper (up to 100× concentration of that in the TMS) were augmented to filtered wastewater. As positive controls, AOA and AOB were incubated in the AFM as described above.

Stimulation of AOA by copper augmentation in AOB-enriched mixed liquor was investigated by inoculation of the sludge with washed cells of *N. koreense* (late log phase), which grows at a temperature range (Table S2) comparable to the operation temperature of the municipal WWTPs. Mixed liquor was collected from the aerobic reactor of CJ and used the same day for inoculation (10%, v/v) after adjustment of the concentration of activated sludge (ca. 30 mg L^−1^). Both NH_4_Cl (1 mM) and HEPES (pH 7.4; 3 mM) were supplemented to the mixed liquor. The effect of copper was tested by augmentation of copper (50× concentration of that in the TMS). Allylthiourea (ATU; 20 µM) was added to selectively inhibit growth of AOB (and possibly comammox) in activated sludge [2, 16, 22]. ATU is a weak Cu^2+^-chelating agent and is a mechanism-based inhibitor [27, 28] selectively inhibiting at the applied concentration the ammonia monooxygenase (AMO) of AOB [29, 30]. In order to measure nitrification by AOB (and potentially comammox) in the activated sludge, the mixed liquor was incubated without ATU. The mixed liquor was incubated at 25 °C in a shaking incubator at 150 rpm. At the start of the experiment and at the point of complete ammonia depletion, mixed liquor samples were collected and suspended solids were harvested by centrifugation at 1000 × *g* for 20 min. The pellet was frozen at −70 °C before extraction of DNA and real-time PCR quantification of bacterial and archaeal *amoA* gene copies.

### Quantification of amoA genes

DNA was extracted from the suspended solids after pelleting by centrifugation at 4000 × *g* for 30 min using the Soil DNA kit (GeneAll, Korea). From these extracts, 10–20 ng of DNA was used for quantification of *amoA* gene copies using a MiniOpticon real-time PCR detection system (Bio-Rad Laboratories, Hercules, CA) and Opticon Monitor Software version 3.1 (Bio-Rad Laboratories, Hercules, CA). The iQ SYBR Green Supermix (Bio-Rad, USA) and specific PCR primers (Table S4) were used for amplification. Dilution series of target DNA sequence were included for every real-time PCR for preparing a standard curve as described previously [25]. Amplification efficiencies ranged from 87 to 102% (*R*^2^ values ≥ 0.98) for AOA *amoA* genes, and from 85 to 97% (*R*^2^ values ≥ 0.98) for AOB *amoA* genes, respectively.

### Ultraviolet (UV) light treatment of wastewater

UV light treatment was used for photolytic decomposition of metal-complexing organic carbon compounds in the wastewater samples. The filtered wastewater of the domestic WWTP CJ was treated in quartz tubes by UV light from four low-pressure mercury lamps (48 W). Prior to the experiment, the quartz tubes were soaked for 24 h with 1 M HCl and rinsed three times with Milli-Q water. After UV treatment the filtered wastewater was supplemented with NH_4_Cl (1 mM) and HEPES (pH 7.4; 1 mM). Copper was augmented at 0×, 0.3×, and 10× concentrations of that contained in the TMS. Cells of *N. viennensis* at late log phase were washed and inoculated (1%, v/v). Cell-free wastewater without UV treatment was used as a control.

### Analysis of concentrations of metals and dissolved organic carbon (DOC) in filtered wastewater

To prepare wastewater for metal and DOC analysis from mixed liquors, the liquid phase was separated from suspended solids by centrifugation at 4000 × *g* for 30 min and filtered using 0.2 µm pore-size PES membranes that were prewashed with 1 M HCl and rinsed with Milli-Q water. After acid treatment of the filtered wastewaters with pure HNO_3_ to a final concentration 2% (v/v), the total concentrations of divalent metal ions in wastewater were analyzed by inductively coupled plasma mass spectrometer (Agilent 7500c, Japan). The DOC concentrations of filtered wastewater samples were determined in triplicate using a TOC analyzer (Shimadzu; TOC-LCSH, Japan) following the manufacturer’s instructions.

### Metal speciation calculations

Concentrations of copper and iron species in the AFM containing various total concentrations of histidine were calculated using MINEQL+ 5.0 (Environmental Research software) with the stability constants for metal-histidine complexes in the model’s thermodynamic database [31]. Total concentrations of each component in the model were as listed in Table S3, except for total histidine, which was varied from 0.5 μM to 5 mM, and ammonia, which was set at 1 mM. Calculations were performed by specifying oxic conditions, without surface complexation, at pH 7.0, a total carbonate concentration of 2 mM, and a closed system.

### Putative genes involved in metal transport

The predicted protein-coding sequences of AOA (*N. koreense* MY1*, Ca*. N. chungbukensis MY2, *Ca*. N. oleophilus MY3, *N. viennensis* EN76) and AOB (*N. europaea* ATCC 19718) were retrieved from the National Center for Biotechnology Information RefSeq database (released in July 2018) [32]. Orthologous genes shared between genomes of these ammonia oxidizers was obtained by using OrthoFinder with default parameters [33]. For screening genes related to metal transport, each orthologous gene was classified according to the transporter classification database [34]. Protein domains of screened genes were annotated locally with Pfam database using pfam_scan.pl (ftp://ftp.ebi.ac.uk/pub/databases/Pfam/Tools/) (version 32.0) [35].

## Results

### Wastewaters inhibit growth of AOA but not AOB

**Fig. 1:**
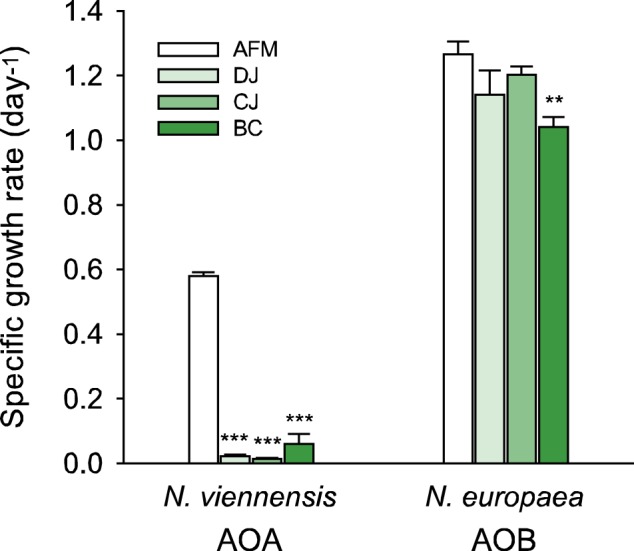
Growth of *N. viennensis* and *N. europaea* in sterile-filtered wastewater of nitrifying reactors from three municipal WWTPs. Cells (1%, v/v) were washed by centrifugation and subsequently inoculated into filtered wastewater (DJ, Daejeon; CJ, Cheongju; BC, Bucheon) amended with ammonium (see more details in “Materials and methods” section). The specific growth rates of both strains in artificial freshwater medium (AFM) were used as positive controls. Error bars represent standard deviation for *n* ≥ 3 biological replicates. Significance of differences between control and filtered wastewaters for each strain was determined by the Student’s *t* test (**p < 0.01 and ***p < 0.005)

Activated sludge samples obtained from aerobic nitrifying bioreactors from three Korean metropolitan municipal WWTPs (Table 1) in DJ, CJ, and BC each contained between three and four orders of magnitude fewer *amoA* gene copies of AOA than AOB (Fig. S1). In order to assess whether growth of AOA and AOB may be inhibited differentially by wastewater from the aerobic reactors, we investigated the growth of an AOA strain (*N. viennensis* EN76) and an AOB strain (*N. europaea* ATCC 19718) (Table S2) in sterile-filtered wastewater from these three WWTPs amended with 1 mM ammonium (Fig. 1). Our results showed that growth of *N. viennensis* was significantly inhibited by all three wastewaters compared with growth in AFM (AFM; see Table S3) with 1× TMS (see Table S3), whereas *N. europaea* grew equally well in wastewaters and AFM with TMS amendment.

### Effect of organic compounds on AOA growth

**Fig. 2:**
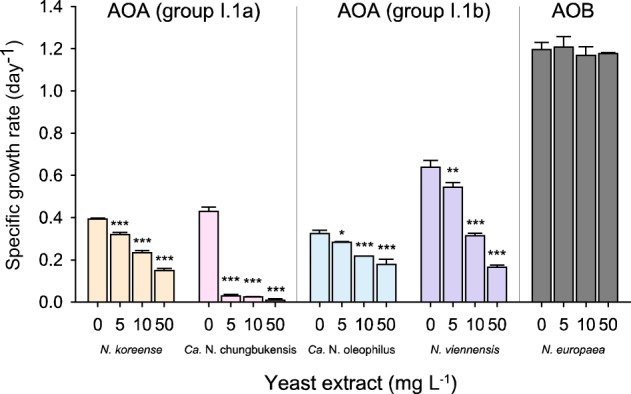
Inhibitory effects of yeast extract on growth of AOA and AOB. Four AOA strains (*N. koreense*, *Ca*. N. chungbukensis, *Ca*. N. oleophilus, and *N. viennensis*) and one AOB strain (*N. europaea*) were inoculated in AFM with 1× TMS amended with various concentrations of yeast extract (0, 5, 10, and 50 mg L^−1^) and the specific growth rate was determined. Error bars represent standard deviation for n ≥ 3 biological replicates. For each strain, significance of differences between growth rates in the control and the yeast extracted amended media was determined by the Student’s *t* test (*p < 0.05, **p < 0.01, and ***p < 0.005)

Because inhibition of AOA strains even by low concentrations of organic compounds was previously observed (Table S1) and wastewater contains relatively high amounts of organic matter, we examined differential growth inhibition of AOA and AOB by complex mixtures of organic compounds (i.e., yeast extract, peptone, and tryptone) and several defined organic compounds. Four AOA strains, belonging either to group I.1a or I.1b of the *Thaumarchaeota*, and the AOB *N. europaea* (Table S2), were individually inoculated into AFM amended with TMS that was supplemented with different concentrations of yeast extract. Increasing concentrations of yeast extract from 0 to 50 mg L^−1^ resulted in decreased specific growth rates (μ) for all tested AOA strains (Fig. 2). *Ca*. N. chungbukensis MY2 was the most sensitive AOA strain and was strongly inhibited (93%) even after addition of 5 mg L^−1^ yeast extract. Amendments with 50 mg L^−1^ yeast extract reduced the specific growth rates of the AOA strains *N. koreense* MY1, *Ca*. N. oleophilus MY3, and *N. viennensis* to 38, 55, and 26% of those in the absence of yeast extract, respectively. In contrast, growth of the AOB strain *N. europaea* was uninhibited at all tested yeast extract concentrations. Taken together, these results indicate that organic compounds present in yeast extract have differentially inhibitory effects on AOA and AOB. In an attempt to identify specific organic compounds within the complex mixtures of organic compounds that inhibit AOA, we tested the effects of various amino acids and organic acids (0.5 mM) on the growth of the AOA and AOB strains in AFM (Fig. S2). Although tryptone, peptone, and humic acids (i.e., humate) inhibited the growth of the AOA strains significantly, *N. europaea* was only partially inhibited by tryptone. At the tested concentration many organic compounds showed no inhibitory effect on any strain, while aspartate inhibited AOA more than the AOB; cysteine and histidine were strongly inhibitory to all tested AOA and AOB strains.

### Inhibitory organic carbon complexes with copper

Because the inhibitory organic acids and amino acids bind metals strongly [36–38], we hypothesized that the observed inhibition of AOA, and partial inhibition of *N. europaea*, was a result of trace metal nutrient limitation. To test this hypothesis, TMS was augmented up to 40× in the AFM and the inhibition experiments were repeated with *N. viennensis* as a test strain and histidine, yeast extract, tryptone, and peptone as inhibitory substances. Interestingly, the inhibitory effects of all compounds on *N. viennensis* could significantly (73–89%) be mitigated by augmentation of trace metals in the medium (Fig. S3). Higher concentrations of trace metals (20× TMS) were required for mitigation of the inhibitory effects of histidine compared with those caused by the complex mixtures of organic compounds for which addition of 2× TMS was sufficient (Fig. S3).

**Fig. 3:**
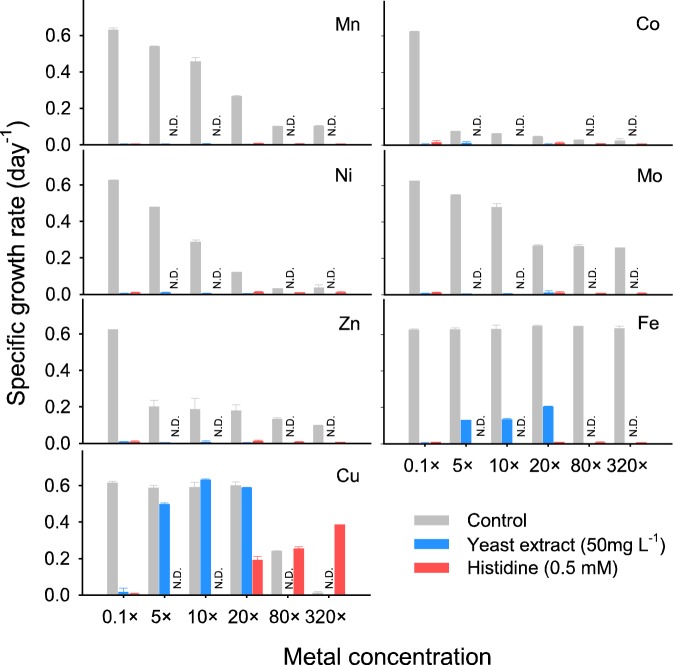
Influence of metal additions on growth restoration of *N. viennensis* inhibited by organic compounds. Trace metal salts (Mn^2+^, Co^2+^, Ni^2+^, Mo^2+^, Zn^2+^, Fe^3+^, and Cu^2+^) in the TMS were individually augmented into AFM amended with 50 mg L^−1^ yeast extract or 0.5 mM histidine. Fe^3+^ was added as iron(III) citrate. Incubations of AFM without supplementation of organic compounds was used as a control. Metal augmentation is indicated as multiples of the concentration in TMS. Error bars represent one standard deviation for *n* ≥ 3 biological replicates. N.D. indicates no data

In order to reveal which specific trace metals become limiting for AOA in the presence of metal-complexing organic compounds in the growth medium, growth experiments with *N. viennensis* were performed in the AFM to which different concentrations of individual trace metals (Mn, Co, Ni, Mo, Zn, Fe, and Cu) were added in the presence of histidine or yeast extract (Fig. 3) at a concentration that fully inhibited growth in the AFM. Whereas increasing the concentration of Mn, Co, Ni, Mo, and Zn did not alleviate inhibition of *N. viennensis* by the tested organic compounds, copper augmentation mitigated the inhibitory effects of histidine and yeast extract (320× and 10× concentration of that in the TMS leading to ~3.75 × 10^−6^ M and ~1.17 × 10^−7^ M Cu in AFM, respectively). As expected from the results shown in Fig. S3, higher copper concentrations were required to mitigate the inhibition of histidine (0.5 mM) than of yeast extract (50 mg L^−1^). Augmentation of the medium with iron (up to 320× concentration of that in the TMS; ~2.4 × 10^−3^ M in AFM) did not restore *N. viennensis* growth in the presence of histidine, whereas iron augmentation to 20× concentration of that in the TMS (1.5 × 10^−4^ M in AFM) partially restored *N. viennensis* growth in the presence of yeast extract (~33% of the positive control). Mitigation of inhibition by yeast extract, tryptone, and peptone via copper addition was clearly demonstrated by using *N. viennensis* and “*Ca*. N. chungbukensis” as test strains (Fig. S4).

**Fig. 4:**
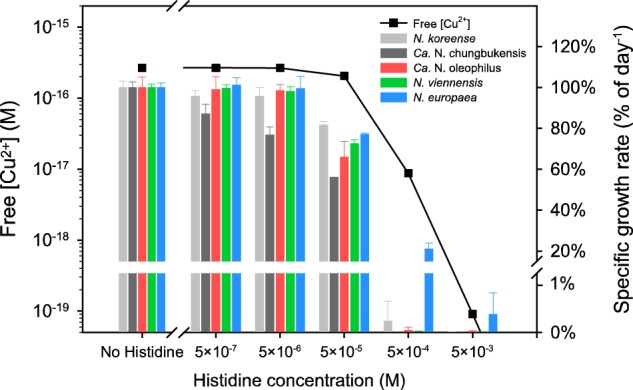
Relationship between free Cu^2+^ concentration and growth rates of AOA and AOB. The free Cu^2+^ concentration in AFM was adjusted by adding varying concentrations of histidine. Free Cu^2+^ concentrations were calculated by MINEQL+. Bars indicate the proportion of specific growth rates of each strain compared with their growth rates in the control experiments in AFM without histidine. Error bars represent standard deviations for *n* ≥ 3 biological replicates

Additional experiments and calculations were performed to estimate the amount of free (unbound) Cu^2+^ and total inorganic copper (Cu′) in the medium necessary for growth of the AOA strains and *N. europaea*. With constant concentrations of inorganic metal-binding ligands and at a constant pH, the concentration of free Cu^2+^ is proportional to that of Cu′, all of which is bioavailable in microorganisms [38, 39]. For the four AOA strains and the AOB strain, growth rates were determined in the AFM supplemented with different amounts of histidine and the amount of free Cu^2+^ was calculated for each medium as a proxy for bioavailable copper with the chemical equilibrium modeling system (MINEQL+) (Fig. 4 and Table S5). Addition of 50 µM histidine that only slightly decreased the free Cu^2+^ concentration from 2.68 × 10^−16^ M in the absence of histidine to 2.06 × 10^−16^ M, decreased the growth rates of the four AOA strains to 56–82% of the rate associated with the control culture without histidine (Fig. 4). At 500 µM (5 × 10^−4^ M) histidine, the predicted free Cu^2+^ concentration in the AFM decreased to 8.76 × 10^−18^ M and growth of all AOA strains was completely inhibited; *N. europaea* growth was reduced to ~21% of the control rate without histidine. Consistent with the results for yeast extract exposure (Fig. 2), *Ca*. N. chungbukensis was the most sensitive strain, inhibited significantly even by the presence of 5 µM histidine (2.67 × 10^−16^ M free Cu^2+^).

### Copper availability in wastewater of aerobic nitrifying reactors

**Fig. 5:**
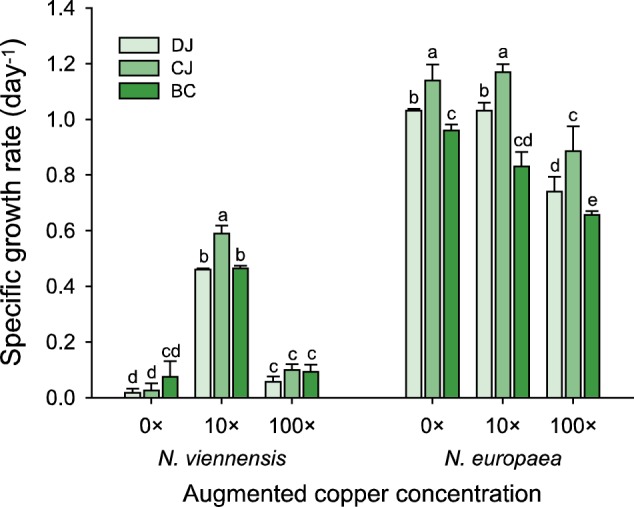
Effect of copper augmentation on growth of *N. viennensis* and *N. europaea* in filtered wastewater. Cells were harvested, washed, and inoculated into sterile-filtered wastewater supplemented with 1 mM ammonium. Copper augmentation is indicated as multiples of the concentration in TMS. Error bars represent standard deviation for *n* ≥ 3 biological replicates. Significant differences between treatments in each strain are indicated by different letters (Two-way ANOVA, Tukey’s test, *p* *<* 0.001)

Based on results for AOA grown in the presence of organic matters and metals, we hypothesized that growth inhibition of AOA in sterile-filtered wastewater from the three municipal WWTPs (Fig. 1) was also due to copper limitation. To test this, the wastewater growth experiment was repeated with filtered aeration basin samples that were amended with copper. In all cases, growth of *N. viennensis* was greatly stimulated (up to ~75–95% of the AFM growth rate) by augmentation with copper concentrations that were tenfold higher than present in AFM (1.17 × 10^−7^ M in the wastewater; Fig. 5). Wastewater-associated growth stimulation by copper was observed for all four AOA strains, although the effect of the copper concentrations on growth stimulation differed for each strain (Fig. S5). In contrast, growth of the AOB strain *N. europaea* was uninhibited by sterile-filtered wastewater (Figs. 1 and 5) and copper augmentation did not stimulate growth (Fig. S5).

After UV light treatment, DOC concentrations of the three filtered wastewaters decreased substantially (Table 1) and growth of *N. viennensis* in the UV-treated filtered wastewater was observed without copper augmentation (Fig. S6), indicating copper release from organic complexes. Based on these findings, we hypothesized that, in wastewater treatment systems where AOA are relatively abundant, copper is not limiting. To test this hypothesis, *N. viennensis* was grown in filtered wastewater samples from an oil refinery WWTP and two RBCs that were reported to contain high abundances of thaumarchaeotes [12, 14] (Table 1), in the presence and absence of added copper (Fig. S7). Interestingly, *N viennensis* could grow well in the filtered wastewater from these plants without copper addition and growth rates were comparable to those in AFM. Addition of copper to the filtered wastewater samples from AOA-dominated plants did not increase the growth rates of *N. viennensis* (Fig. S7).

**Fig. 6:**
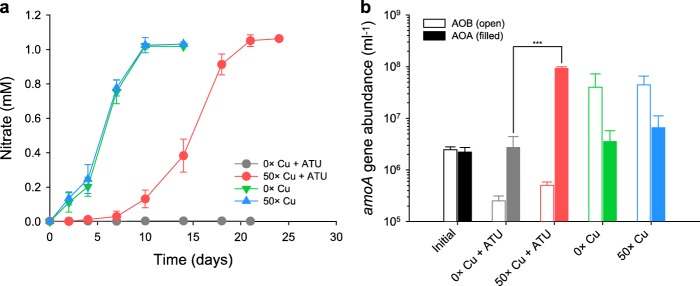
Effect of copper augmentation on growth of *N. koreense* in unfiltered mixed liquor from a nitrifying reactor of a municipal WWTP. Washed cells of *N. koreense* (10%, v/v) were inoculated into the AOB-enriched mixed liquor from the municipal WWTP CJ. To investigate the effect of copper augmentation, 50× concentrations of the copper contained in the TMS was added to incubations as indicated. Allylthiourea (ATU, 20 µM) was used for inhibiting AOB in the activated sludge, respectively. All samples were supplemented with 1 mM ammonium. **a** During ammonia oxidation, a concomitant increase of nitrate was observed. **b** At the start of the experiment and after ammonia depletion, archaeal and bacterial *amoA* gene abundances were measured. Error bars represent standard deviation for *n* ≥ 3 biological replicates. Significance of differences was determined by the Student’s *t* test (***p < 0.005)

Finally, we investigated whether AOA could perform ammonia oxidation in unfiltered activated sludge of a municipal WWTP in the presence of sufficient copper. For this purpose experiments with 20 µM ATU as specific inhibitor of AOB in WWTPs [16, 22] were performed. As expected, no ammonia oxidation was observed in this AOB-dominated sludge in the presence of ATU (Fig. 6). In parallel, activated sludge from the same plant was inoculated with washed cells of *N. koreense* in order to achieve comparable AOB and AOA numbers at the start of the incubations (Fig. 6). Without addition of ATU, only AOB were growing during oxidation of 1 mM of added ammonium, which was completely oxidized within 10 days regardless of copper addition. However, only the added AOA was strongly growing in the presence of ATU and copper (Fig. 6).

## Discussion

Pristine ecosystems, such as most soils and marine environments, are dominated by AOA rather than AOB [3, 4], whereas the opposite relative abundances are commonly observed for engineered environments, including activated sludge systems of most municipal WWTPs [9–11] and in eutrophic lakes [6, 8] and fertilized terrestrial environments [7]. Because several AOA strains were reported to be inhibited in pure culture by organic compounds (Table S1), we hypothesized that organic matter may be a key factor affecting the activity and competitive success of AOA populations in natural and man-made environments, including WWTPs. Consequently, we investigated the effect of various organic compounds on the growth of AOA and AOB, exploring possible mechanisms connected to the presence of organic matter that could be responsible for the low abundance commonly reported for AOA in the activated sludge of municipal WWTPs. We demonstrate that the inhibition of AOA by organic matter is linked to complexation of copper, which greatly limits the bioavailability of Cu^2+^ (Figs. 3 and 4, S4) and is responsible for growth inhibition of AOA in filtered wastewater from nitrifying reactors of municipal WWTPs (Figs. 5 and S5). Furthermore, we show that copper supplementation strongly supported growth of AOA in the presence of inhibitory organic compounds in standard media (Figs. 3 and S4) and in filtered wastewater from municipal treatment plants (Figs. 5 and S5). In the presence of elevated copper concentrations, AOA even grew in unfiltered activated sludge from municipal WWTPs and maintained ammonia oxidation when AOB were selectively inhibited by ATU (Fig. 6).

The extent to which organic compounds limit the bioavailability of essential divalent metals varies with the stability constants (log *K*) for various divalent metal–organic complexes [36, 38]. Thus, a wide range of organic acids, amino acids, and peptides with the potential to form metal complexes might be involved in the inhibition of AOA growth. Jung et al. [25] found that most of the dipeptides that are inhibitory to the AOA strain “*Ca*. N. oleophilus” have high metal complexation potential. Organic complexation is especially important for copper(II) (Cu^2+^), the dominant form of dissolved copper in oxic waters [36, 40], which is at the top of the Irving–Williams Series [41] referring to the relative stabilities of complexes formed by transition metals. Accordingly, among metal cations, Cu^2+^ typically has the highest affinity for most environmental organic ligands [42]. Among common organic compounds found in wastewater and natural water [43, 44], cysteine and histidine form very stable complexes with Cu^2+^ and are highly inhibitory to AOA. Other organic compounds from fresh and partially decomposed (e.g., amino acids, sugars, and peptides) and well-decomposed (e.g., humic acids) organic matter are also capable of forming strong complexes with free Cu^2+^ [42, 45]. Thus, most dissolved Cu^2+^ (98–99%) in natural and engineered aquatic environments is complexed with organic matter [36, 40].

Copper is required for ammonia oxidation by both AOA and AOB due to the presence of essential mono- and di-nuclear copper centers of the key enzyme AMO [46, 47]. In addition, copper is essential for the functioning of the nitrite reductase NirK that is encoded by most AOA and AOB and plays important roles for their physiologies [48, 49]. However, the electron transport system of AOB contains some iron (Fe)-dependent metalloenzymes and proteins [the multiheme cytochrome c hydroxylamine oxidoreductase (HAO) and other c-type cytochromes] that are not encoded in known AOA genomes [50]. In contrast, AOA are postulated to possess a more copper-based electron-transfer system inferred from (i) the numerous genes encoding copper-containing proteins such as multi-copper oxidases and proteins with small Cu-binding plastocyanin-like domains in AOA genomes [25, 51, 52] and (ii) their detection in AOA proteomes [51, 53, 54], although the iron-containing electron carrier protein ferredoxin is also encoded and expressed in AOA [48, 51, 53, 54]. Thus, the strong inhibition of AOA by copper-complexing organic compounds is most likely caused by impaired function of copper-containing proteins in AOA which are essential for growth. In addition, different affinities of the copper uptake systems of AOB and AOA might contribute to the increased sensitivity of AOA to inhibition by copper-complexing organic matter (see discussion in Supplementary information and Table S6). Clearly, further studies are needed for revealing the mechanism(s) causing the high sensitivity of AOA to limitations in bioavailable copper.

Complexation of copper with organic matter in activated sludge was demonstrated in several previous studies that focused on copper toxicity for microbially mediated processes [55]. Elevated copper concentrations were required to inhibit nitrification in activated sludge over those needed for achieving the same effects in pure cultures of AOB; [56] the amount of copper required to inhibit nitrification was proportional to the sludge biomass [57]. Limitation of copper bioavailability may be a factor reducing the activity of archaeal nitrification in seawater [26] and copper deficiency in biological filters of drinking water treatment systems limits ammonia oxidation [58]. Concentrations of total dissolved copper in filtered wastewater from the aerobic reactors (Table 1) were at least tenfold higher than those in the AFM (Table S3), which indicates that the bioavailable form of Cu^2+^ is significantly depleted by the presence of organic matter in these systems. This is supported by the observation of AOA growth in the filtered wastewaters without copper augmentation after degradation of organic matter by UV treatment (Table 1 and Fig. S6). It is noteworthy that in the presence of copper, but absence of ATU, AOB still outcompete AOA in the activated sludge (Fig. 6). Because this might reflect the addition of a high ammonia concentration in this batch experiment, it would be worthwhile to perform continuous culture experiments with municipal activated sludge at elevated copper concentrations to investigate whether AOA can then outcompete AOB. Indeed, Srithep et al. [16] demonstrated that the activity of AOA in sludge of nitrifying reactors can be promoted by addition of various trace metals, including copper. Interestingly, growth of AOA strains in our study was observed in filtered wastewater from plants with a high in situ AOA abundance without augmentation of copper [12, 22], suggesting that copper limitation did not have the same impact on AOA as in the municipal WWTPs. The wastewater samples from the plants with high AOA abundances contained relatively high total copper and iron concentrations, whereas measured DOC concentrations were not significantly different from the municipal wastewaters (Table 1). It should also be noted that salicylate, which has a high complexation potential for iron [59], was not inhibitory to the growth of the four AOA strains (Fig. S2). We conclude that iron availability did not impact the histidine-limited growth of the AOA in the presence of organic compounds in our experiments (Fig. 4) because there were no significant changes in free (~1.95 × 10^−22^ M) or kinetically labile iron (Fe′) concentrations (data not shown). In addition, a recent finding suggests that the AOA *Nitrosopumilus maritimus* SCM1 can utilize siderophore-bound iron through a reductive uptake pathway [60]. However, competition between copper and iron for metal-complexing humic substances was important for bioavailability of both metals to microorganisms in estuarine waters [61]. Consistently, iron augmentation partially alleviated inhibition of *N. viennensis* by yeast extract (Fig. 3), which indicates that copper availability to AOA might be influenced by both organic ligands and the iron concentration in wastewaters.

Inhibition of growth by organic compounds has been widely observed in diverse autotrophs, and even oligotrophic heterotrophs, which play key roles in biogeochemical processes in various environments (Table S1). Nonetheless, systematic investigations of organic compound inhibition of microbes are rare. The mechanism of organic compound inhibition of AOA growth caused by metal complexation and reduced metal bioavailability as a nutrient that we demonstrate may also be relevant for explaining organic compound inhibition of various other autotrophic and oligotrophic heterotrophic microorganisms. Thus, our findings might have broader implications for understanding the activity and composition of a wide array of environmentally relevant bacteria and archaea.

Overall, we demonstrate different sensitivities of AOA and AOB strains to organic matter and identified the reduction of copper bioavailability by metal-complexing organic compounds as the mechanism for AOA growth inhibition in municipal activated sludge. Given the high dependency of AOA on copper, this inhibitory mechanism could provide a basis for developing approaches for modulating the composition of nitrifying communities in terrestrial, aquatic, and engineered environments. Keeping in mind that AOA have a lower N_2_O yield per mol ammonium oxidized than AOB [62], contribute to transformation of micropollutants [63], have greater adaptability to live in extreme environments than AOB [20], and possess a higher substrate affinity than AOB [18, 19], such modulations might help to reduce greenhouse gas emissions from WWTPs and agricultural soils and could contribute to improved micropollutant transformation and ammonia effluent concentrations in various sewage treatments.

## Supplementary information

supplementary information
