# Supplementary material for: Archaeal nitrification is constrained by copper complexation with organic matter in municipal wastewater treatment plants
Source: ISME J. 2019 Oct 17;14(2):335–46. doi: 10.1038/s41396-019-0538-1 (PMC6976641; doi:10.1038/s41396-019-0538-1)
Supplement: Supplementary file 1 — supplementary information [file 41396_2019_538_MOESM1_ESM.docx]

**Supplementary Information**

**Archaeal nitrification is constrained by copper complexation with organic matter in municipal wastewater treatment plants**

Joo-Han Gwak^a,1^, Man-Young Jung^b,1^, Heeji Hong^a^, Jong-Geol Kim^a^, Zhe-Xue Quan^c^, John R. Reinfelder^d^, Emilie Spasov^e^, Josh D. Neufeld^e^, Michael Wagner^b,f^, and Sung-Keun Rhee^a,2^

^a^Department of Microbiology, Chungbuk National University, 1 Chungdae-ro, Seowon-Gu, Cheongju 28644, South Korea; ^b^Centre for Microbiology and Environmental Systems Science, Department of Microbiology and Ecosystem Science, Division of Microbial Ecology, University of Vienna, Althanstrasse 14, A-1090 Vienna, Austria; ^c^Ministry of Education Key Laboratory for Biodiversity Science and Ecological Engineering, Institute of Biodiversity Science, School of Life Sciences, Fudan University, Shanghai, China; ^d^Department of Environmental Sciences, Rutgers University, New Brunswick, NJ, 08901, USA; ^e^Department of Biology, University of Waterloo, Waterloo, N2L 3G1 ON, Canada; ^f^Center for Microbial Communities, Department of Chemistry and Bioscience, Aalborg University, Fredrik Bajers Vej 7H, 9220 Aalborg, Denmark

^1^J.-H.G and M.-Y.J contributed equally to this work.

^2^To whom correspondence should be addressed. E-mail: [rhees@chungbuk.ac.kr](mailto:rhees@chungbuk.ac.kr)

**This document includes:**

Supplementary discussion

Supplementary Tables S1 to S6

Supplementary Figures S1 to S7

Supplementary References

**Supplementary discussion**

**Different repertoires of copper uptake systems between AOB and AOA strains**

Prokaryotes have multiple systems for copper transport to supply copper to copper-containing proteins and enzymes [[1](#_ENREF_1)]. For high affinity copper uptake, copper complexation by the chalkophore methanobactin is a key mechanism used by methanotrophs [[2](#_ENREF_2)]. A TonB-dependent transporter is responsible for uptake of the copper-methanobactin complex by *Methylosinus trichosporium* OB3b [[3](#_ENREF_3)]. Siderophores are a diverse group of specialized iron-complexation metabolites that function to supply iron to cells. Because recent evidence suggests that iron siderophore tightly bind copper, copper-siderophore complexes could be recognized and transported through the siderophore-specific transport machinery [[4](#_ENREF_4), [5](#_ENREF_5)], implying a promiscuous nature for these organic ligands and transporters. Genes involved in the synthesis of known chalkophores and siderophores are absent in sequenced AOA genomes [[6-8](#_ENREF_6)], whereas citrate was proposed as the only possible siderophore candidate for *N. europaea* [[9](#_ENREF_9)]. Consistently, Amin et al. [[10](#_ENREF_10)] and Shafiee et al. [[11](#_ENREF_11)] demonstrated that no strong organic ligands, such as chalkophores or siderophore, were produced by the marine AOA *Nitrosopumilus maritimus* SCM1 even under copper-limiting conditions. There are no putative genes encoding TonB-dependent receptors for metal-complexed organic ligands in AOA genomes. In contrast, various candidate genes encoding TonB-dependent receptors for metal-complexed organic ligands are encoded in the genome of *N. europaea* (Table S6). In fact, growth of *Nitrosomonas* sp. RA was supported by siderophores produced by an associated heterotroph, possibly via TonB-dependent receptors [[12](#_ENREF_12)]. Thus, in the presence of organic ligands produced by other microorganisms, AOB may take up organic complexes of Cu^2+^, which could give AOB an advantage over AOA in eutrophic environments where bioavailable Cu^2+^ is limited by organic matter complexation.

Different degree of inhibition of organic matter to different AOA strains might be linked to different repertoires of transport systems for free and kinetically labile inorganic complexes of Cu^2+^ (Cu'), whose concentration in a complex mixture can be estimated based on a chemical equilibrium modeling system (Fig. 4 and Table S5). We screened the genomes of the four AOA strains for putative dedicated copper transporters and other transporters that might be involved in copper transport. Genes encoding a cation diffusion facilitator (CDF) [[13](#_ENREF_13)], metal ion (Mn^2+^-iron) transporter (Nramp) [[14](#_ENREF_14)], an ABC-type Zn uptake system [[15](#_ENREF_15)], a copper resistance (CopD) family [[16](#_ENREF_16)], a member of the zinc (Zn^2+^)-iron (Fe^2+^) permease (ZIP) family [[17](#_ENREF_17)], and a member of the P-type ATPase (P-ATPase) superfamily [[18](#_ENREF_18)] were found to be encoded in at least one of the four tested AOA strains (Table S6). Even though additional research is necessary to reveal whether the above listed genes are actually involved in copper transport, differences in the putative copper transporter repertoire encoded by each of the tested AOA strains (Table S6) might reflect heterogeneity among these AOA with respect to their affinity for copper. In addition, cells of *N. maritimus* passively concentrate Cu^2+^ onto their surfaces, which could compete with dissolved organic carbon for Cu^2+^ complexation [[19](#_ENREF_19)]. Comparative genomics of AOA reveals possible diversity of cell surface structures. For examples, the cell wall S-layer proteins are very diverse among AOA members [[7](#_ENREF_7)] and genomic islands predicted to be involved in cell surface modification are widespread [[20](#_ENREF_20)]. In addition, *N. viennensis* and "*Ca.* N. oleophilus” are predicted to produce extracellular polymeric substances [[6](#_ENREF_6), [7](#_ENREF_7)]. The above discussed traits likely contributed to the observed strain-specific differences in response to copper additions in our experiments (e.g., Fig. S5) and might influence the abundances, distributions, and activities of AOA and AOB in their natural environments.

**Table S1. Inhibition of autotrophs and oligotrophic marine bacteria by organic compounds**

| **Species** | **Organic substrates** | **Concentration** | **Reference** |
| --- | --- | --- | --- |
| **Ammonia-oxidizing archaea** |  |  |  |
| *Nitrosarchaeum koreense* MY1 | histidine, aspartate, cysteine, tryptophan, citrate, malate | 0.5 mM | Jung et al. [[21](#_ENREF_21)] |
|  | peptone, yeast extract | 0.05 g L^-1^ |  |
| *Nitrosopumilus maritimus* SCM1 | citrate, acetyl-coa, lactate, salicylate, tryptophan, trimethylamine, methylamine | 0.1 mM | Qin et al. [[22](#_ENREF_22)] |
|  | xylan, casein peptone, bovine serum albumin, humic acid | 0.05 g L^-1^ |  |
|  | yeast extract | 0.005 g L^-1^ |  |
| *Nitrosocaldus yellowstonii HL 72* | yeast extract | 0.0002 g L^-1^ | de la Torre et al. [[23](#_ENREF_23)] |
|  | acetate | 2 mM |  |
| *Nitrosotalea devanaterra* *Nd1* | pyruvate. citrate, α-ketoglutarate, succinate, fumarate, malate, oxaloacetate | 100 mM | Lehtovirta-Morley et al. [[24](#_ENREF_24)] |
| *Nitrosocosmicus oleophilus* MY3 | citrate, malate, arginine, histidine, aspartate, glutamate, cysteine, tyrosine, tryptophan | 1 mM | Jung et al. [[25](#_ENREF_25)] |
|  | peptone, yeast extract, casamino acids | 0.5 g L^-1^ |  |
| **Ammonia-oxidizing bacteria** |  |  |  |
| *Nitrosomonas* sp. N3 | tryptone soya broth, nutrient broth, yeast extract, peptone | 0.01 g L^-1^ | [Macfarlane and Herbert [26]](#_ENREF_26) |
| *Nitrosomonas europaea* | valine, arginine, lysine, histidine, threonine, methionine, glutamine | 0.004 g L^-1^ | [Clark and Schmidt [27]](#_ENREF_27) |
| **Nitrite-oxidizing bacteria** |  |  |  |
| *Nitrospira moscoviensis* | yeast extract, meat extract, peptone | 0.75 g L^-1^ | Ehrich et al. [[28](#_ENREF_28)] |
| **Iron-oxidizing bacteria** |  |  |  |
| *Leptospirillum*-like bacteria | yeast extract | 0.5 g L^-1^ | [Vardanyan and Akopyan [29]](#_ENREF_29) |
| *Ferrobacillus ferrooxidans* | peptone | ND | Braley et al. [[30](#_ENREF_30)] |
| **Sulfur-oxidizing bacteria** |  |  |  |
| *Thiobacillus neapolitanus* | phenylalanine, histidine, methionine, threonine | 0.3 mM | [Johnson and Vishniac [31]](#_ENREF_31) |
| **Methane-oxidizing bacteria** |  |  |  |
| *Acidimethylosilex fumarolicum* | acetate, malate, succinate, formate, formaldehyde, yeast extract | 1 g L^-1^ | Pol et al. [[32](#_ENREF_32)] |
| *Methylococcus capsulatus* | threonine, leucine, histidine, glycine, proline, | 0.1-0.2 g L^-1^ | Eroshin et al. [[33](#_ENREF_33)] |
|  | lysine, methionine, glutamate, tryptophane, arginine hydroxyproline, tyrosine, citrate, casamino acids, isoleucine, fumarate, succinate, acetate, 2-oxoglutaric acid | 1-5 g L^-1^ |  |
| **Phototrophs** |  |  |  |
| *Desmodesmus communis*, *Chrococcus minutus* | natural organic matter | 0.000025 g L^-1^ | [Heinrich [34]](#_ENREF_34) |
| *Microcystis aeruginosa*, *Planktothrix agardhii* | dissolved organic matter | 0.262 mM | Nagai et al. [[35](#_ENREF_35)] |
| **Oligotrophic heterotrophs** |  |  |  |
| SAR11 | peptone | 0.01 g L^-1^ | Rappé et al. [[36](#_ENREF_36)] |
| Bacterioplankton | amino acids (casein hydrolysate) | 0.005 g L^-1^ | Button et al. [[37](#_ENREF_37)] |

**Table S2. Properties of AOA and AOB strains used in this study**

| **Organism** | **Phylogenetic group** | **Specific growth rate (day^-1^)** | **Total cell counts after cultivation in AFM (cells ml^-1^)**^a^ | **pH range supporting growth**  **(optimum)** | **Temperature range supporting growth (°C)**  **(optimum)** |
| --- | --- | --- | --- | --- | --- |
| *Nitrosarchaeum koreense* MY1 | TG^b^ I.1a | 0.39 | 2.7 × 10^8^ | 6.0−8.0 (7.0) | 15−30 (25) |
| *Ca.* Nitrosotenuis chungbukensis MY2 | TG I.1a | 0.42 | 3.2 × 10^8^ | 6.0−8.5 (7.0) | 25−40 (30) |
| *Ca.* Nitrosocosmicus oleophilus MY3 | TG I.1b | 0.31 | 2.8 × 10^6^ | 5.5−8.5 (7.0) | 25−30 (30) |
| *Nitrososphaera viennensis* EN76 | TG I.1b | 0.59 | 5.8 × 10^7^ | 6.0−8.5 (7.5)^c^ | 28−47 (42)^c^ |
| *Nitrosomonas europaea* ATCC 19718 | *Betaproteobacteria* | 1.25 | 6.5 × 10^6^ | 6.0−9.0 (7.5) | 20−30 (25) |

^a^ Total cell counts after oxidation of 1 mM ammonia in AFM

^b^ TG, Thaumarchaeotal Group

^c^ Data from Tourna et al. [[38](#_ENREF_38)] and Stieglmeier et al. [[39](#_ENREF_39)]

**Table S3. Composition of the basal mineral salts and trace metals solution (TMS) of artificial freshwater medium (AFM) used in this study**

| **Chemical** |  | **(g** **L^-1^)** | **(M)** |  | **(g** **L^-1^)** | **(M)** |
| --- | --- | --- | --- | --- | --- | --- |
| **Mineral salts** |  |  |  |  |  |  |
| MgCl_2_∙6H_2_O |  | - | - |  | 0.4 | 1.97×10^-3^ |
| KCl |  | - | - |  | 0.5 | 6.71×10^-3^ |
| KH_2_PO_4_ |  | - | - |  | 0.2 | 1.47×10^-3^ |
| NaCl |  | - | - |  | 1 | 1.71×10^-2^ |
| CaCl_2_∙2H_2_O |  | - | - |  | 0.1 | 6.80×10^-4^ |
|  |  |  |  |  |  |  |
| **TMS** |  | ***Stock concentration* (1,000×)** | |  | ***Final concentration* (0.1×) in** **AFM**^*^ | |
| CuCl_2_ 2H_2_O |  | 0.002 | 1.17×10^-5^ |  | 2.0×10^-7^ | 1.17×10^-9^ |
| MnCl_2_ 4H_2_O |  | 0.1 | 5.05×10^-4^ |  | 1.0×10^-5^ | 5.05×10^-8^ |
| H_3_BO_3_ |  | 0.03 | 4.85×10^-4^ |  | 3.0×10^-6^ | 4.85×10^-8^ |
| ZnCl_2_ |  | 0.068 | 4.98×10^-4^ |  | 6.8×10^-6^ | 4.98×10^-8^ |
| CoCl_2_ 6H_2_O |  | 0.19 | 7.98×10^-4^ |  | 1.9×10^-5^ | 7.98×10^-8^ |
| NiCl_2_ 6H_2_O |  | 0.024 | 1.01×10^-4^ |  | 2.4×10^-6^ | 1.01×10^-8^ |
| Na_2_MoO_4_ H_2_O |  | 0.036 | 1.49×10^-4^ |  | 3.6×10^-6^ | 1.49×10^-8^ |
| FeCl_2_ 4H_2_O |  | 1.49 | 7.49×10^-3^ |  | 1.49×10^-4^ | 7.49×10^-7^ |
| Na_2_-EDTA |  | 5.2 | 1.40×10^-2^ |  | 5.2×10^-4^ | 1.40×10^-6^ |

^*^ 0.1**×** TMS was used for the standard AFM unless stated otherwise.

**Table S4. Primers used for real-time PCR quantification of bacterial and archaeal *amoA* genes**

| **Target gene** | **Application** | **Sequence (5′ to 3′)** | **Reference** |
| --- | --- | --- | --- |
| Arch-amoAF | Archaeal *amoA* gene  quantification | STAATGGTCTGGCTTAGACG | Francis et al. [[40](#_ENREF_40)] |
| Arch-amoAR |  | GCGGCCATCCATCTGTATGT |  |
|  |  |  |  |
| amoA1F | amoA gene quantification of betaproteobacterial AOB | GGGGTTTCTACTGGTGGT | Rotthauwe et al. [[41](#_ENREF_41)] and Avrahami et al. [[42](#_ENREF_42)] |
| amoA2R |  | CCCCTCKGSAAAGCCTTCTTC |  |

**Table S5. Copper species in the AFM with different concentrations of histidine**

| **Augmented histidine (M)** | **0^*^** | **5×10^-7^** | **5×10^-6^** | **5×10^-5^** | **5×10^-4^** | **5×10^-3^** |
| --- | --- | --- | --- | --- | --- | --- |
| **Species** | **Concentration (M)** | | | | | |
| Total copper | 1.173×10^-9^ | 1.173×10^-9^ | 1.173×10^-9^ | 1.173×10^-9^ | 1.173×10^-9^ | 1.173×10^-9^ |
| **Cu-His (% of total copper)** | - | **1.111×10^-13^ (0.01%)** | **4.211×10^-12^ (0.36%)** | **2.718×10^-10^ (23.17%)** | **1.135×10^-09^ (96.72%)** | **1.172×10^-09^ (99.94%)** |
| CuHIS | **-** | 7.65×10^-14^ | 7.623×10^-13^ | 5.877×10^-12^ | 2.502×10^-12^ | 2.591×10^-13^ |
| Cu(HIS)_2_ | **-** | 3.462×10^-14^ | 3.449×10^-12^ | 2.659×10^-10^ | 1.132×10^-09^ | 1.172×10^-09^ |
| **Cu-EDTA (% of total copper)** | **1.172×10^-09^ (99.95%)** | **1.172×10^-09^ (99.95%)** | **1.168×10^-09^ (99.61%)** | **9.011×10^-10^ (76.83%)** | **3.843×10^-11^ (3.28%)** | **4.045×10^-13^ (0.03%)** |
| Cu[EDTA]^-2^ | 1.172×10^-9^ | 1.172×10^-9^ | 1.168×10^-9^ | 9.009×10^-10^ | 3.842×10^-11^ | 4.044×10^-13^ |
| CuH[EDTA]^-1^ | 3.708×10^-13^ | 3.708×10^-13^ | 3.695×10^-13^ | 2.849×10^-13^ | 1.215×10^-14^ | 1.279×10^-16^ |
| CuOH[EDTA]^-3^ | 1.173×10^-14^ | 1.173×10^-14^ | 1.168×10^-14^ | 9.009×10^-15^ | 3.842×10^-16^ | 4.044×10^-18^ |
| CuH_2_[EDTA] | 5.877×10^-18^ | 5.876×10^-18^ | 5.856×10^-18^ | 4.515×10^-18^ | 1.926×10^-19^ | 2.027×10^-21^ |
| **Cu' (% of total copper)** | **1.325×10^-15^ (1.36×10^-4^%)** | **1.325×10^-15^ (1.36×10^-4^%)** | **1.321×10^-15^ (1.35×10^-4^%)** | **1.018×10^-15^**  **(1.04×10^-4^%)** | **4.335×10^-17^ (4.44×10^-6^%)** | **4.49×10^-19^ (4.60×10^-8^%)** |
| **Free Cu^2+^ (% of total copper)** | **2.68×10^-16^ (2.28×10^-5^%)** | **2.68×10^-16^ (2.28×10^-5^%)** | **2.67×10^-16^ (2.28×10^-5^%)** | **2.058×10^-16^ (1.75×10^-5^%)** | **8.763×10^-18^ (7.47×10^-7^%)** | **9.075×10^-20^ (7.74×10^-9^%)** |
| CuCO_3_ (aq) | 1.181×10^-15^ | 1.181×10^-15^ | 1.177×10^-15^ | 9.073×10^-16^ | 3.862×10^-17^ | 4×10^-19^ |
| CuOH^+^ | 8.533×10^-17^ | 8.532×10^-17^ | 8.503×10^-17^ | 6.555×10^-17^ | 2.79×10^-18^ | 2.89×10^-20^ |
| CuHCO_3_^+^ | 2.7×10^-17^ | 2.699×10^-17^ | 2.69×10^-17^ | 2.074×10^-17^ | 8.828×10^-19^ | 9.142×10^-21^ |
| CuNH_3_^2+^ | 1.555×10^-17^ | 1.555×10^-17^ | 1.549×10^-17^ | 1.194×10^-17^ | 5.084×10^-19^ | 5.265×10^-21^ |
| CuCl^+^ | 1.279×10^-17^ | 1.279×10^-17^ | 1.275×10^-17^ | 9.826×10^-18^ | 4.183×10^-19^ | 4.332×10^-21^ |
| Cu(CO_3_)_2_^2-^ | 2.38×10^-18^ | 2.379×10^-18^ | 2.371×10^-18^ | 1.828×10^-18^ | 7.781×10^-20^ | 8.058×10^-22^ |
| Cu(OH)_2_ (aq) | 1.714×10^-18^ | 1.714×10^-18^ | 1.708×10^-18^ | 1.317×10^-18^ | 5.606×10^-20^ | 5.806×10^-22^ |
| CuCl_2_ (aq) | 1.336×10^-19^ | 1.336×10^-19^ | 1.331×10^-19^ | 1.026×10^-19^ | 4.369×10^-21^ | 4.524×10^-23^ |
| Cu(OH)_3_^-^ | 3.541×10^-22^ | 3.541×10^-22^ | 3.528×10^-22^ | 2.72×10^-22^ | 1.158×10^-23^ | 1.199×10^-25^ |
| CuCl_3_^-^ | 3.756×10^-23^ | 3.755×10^-23^ | 3.742×10^-23^ | 2.885×10^-23^ | 1.228×10^-24^ | 1.272×10^-26^ |
| CuCl_4_^2-^ | 5.669×10^-27^ | 5.669×10^-27^ | 5.649×10^-27^ | 4.355×10^-27^ | 1.854×10^-28^ | 1.92×10^-30^ |
| Cu(OH)_4_^2-^ | 2.806×10^-28^ | 2.806×10^-28^ | 2.796×10^-28^ | 2.155×10^-28^ | 9.176×10^-30^ | 9.503×10^-32^ |
| Cu_2_(OH)_2_^2+^ | 1.829×10^-28^ | 1.829×10^-28^ | 1.816×10^-28^ | 1.079×10^-28^ | 1.956×10^-31^ | 2.098×10^-35^ |
| **Free Cu^2+^/Cu' (%)** | **16.81%** | **16.81%** | **16.81%** | **16.81%** | **16.81%** | **16.81%** |

^*^ Without supplementation of histidine.

**Table S6. Summary of putative genes involved in copper transport**

| **Transporter** | **TCDB** | **Pfam** | **Functional property** | **AOB** |  | **AOA** | | | | **Reference** |
| --- | --- | --- | --- | --- | --- | --- | --- | --- | --- | --- |
|  |  |  |  | ***N. europaea*** |  | ***N. koreense* MY1** | ***Ca.* N. chungbukensis MY2** | ***Ca.* N. oleophilus MY3** | ***N. viennensis* EN76** |  |
| ***N. europaea specific*** |  |  |  |  |  |  |  |  |  |  |
| Outer Membrane Receptor (OMR) Family | 1.B.14 | PF07715  PF00593 | TonB-dependent outer membrane receptor | CAD84457.1, CAD84547.1, CAD84642.1, CAD84669.1, CAD85000.1, CAD85008.1, CAD85101.1, CAD85451.1, CAD85632.1, CAD86415.1, CAD84460.1, CAD84470.1 |  | - | - | - | - | Chain et al. [[43](#_ENREF_43)] |
|  |  |  |  |  |  |  |  |  |  |  |
| ***AOA specific*** |  |  |  |  |  |  |  |  |  |  |
| Cation Diffusion Facilitator (CDF) Family | 2.A.4 | PF01545  PF16916 | Divalent metal cation (Fe/Co/Zn/Cd) transporter | - |  | EGP93063.1, EGP93350.1 | WP_042685183.1, WP_042686416.1 | ALI34788.1, ALI36283.1, ALI37593.1, ALI35133.1, ALI35167.1 | AIC15463.1, AIC16565.1, AIC15299.1 | Anton et al. [[44](#_ENREF_44)] |
| Metal Ion (Mn^2+^-iron) Transporter (Nramp) Family | 2.A.55 | PF01566 | Divalent metal cation transporter | - |  | EGP93472.1 | - | ALI35789.1 | - | [Nevo and Nelson [14]](#_ENREF_14) |
| ATP-binding Cassette (ABC) Superfamily | 3.A.1 | PF01297 | ABC-type Zn uptake system ZnuABC, Zn-binding component | - |  | EGP94519.1 | WP_052347476.1 | ALI34811.1 | AIC16362.1, AIC15737.1 | Pontel et al. [[45](#_ENREF_45)] |
| Copper Resistance (CopD) Family | 9.B.62 | PF05425 | Copper export protein | - |  | EGP94573.1, EGP94516.1 | WP_042684050.1, WP_042684345.1 | ALI37701.1, ALI34541.1, ALI34322.1 | AIC15548.1, AIC15672.1, AIC15670.1 | [Bondarczuk and Piotrowska-Seget [46]](#_ENREF_46) |
|  |  |  |  |  |  |  |  |  |  |  |
| ***N. europaea and AOA*** |  |  |  |  |  |  |  |  |  |  |
| Zinc (Zn^2+^)-Iron (Fe^2+^) Permease (ZIP) Family | 2.A.5 | PF02535 | Zinc/iron permease | CAD85564.1 |  | EGP93062.1 | WP_042684908.1 | ALI37801.1, ALI37406.1 | AIC14373.1, AIC14242.1 | Wintz et al. [[47](#_ENREF_47)] |
|  |  |  |  |  |  |  |  |  |  |  |
| ***Some AOA and***  ***N. europaea*** |  |  |  |  |  |  |  |  |  |  |
| P-type ATPase (P-ATPase) Superfamily | 3.A.3 | PF00403  PF00122  PF00702 | Copper exporting ATPase | CAD84930.1 |  | - | WP_042687657.1 | ALI35899.1 | AIC15525.1 | Argüello et al. [[18](#_ENREF_18)] |

**Fig. S1. Abundances of AOA and AOB in nitrifying activated sludge of three Korean municipal WWTPs.** Quantitative PCR of *amoA* genes was performed with primer pairs specific for AOB and AOA, respectively (Supplementary Table S4). Error bars represent mean standard deviation for n ≥ 3 technical replicates. Please note that the activated sludge might contain complete ammonia oxidizing microbes (comammox, Daims et al. [[48](#_ENREF_48)]) in addition (not tested here as not relevant for this study).


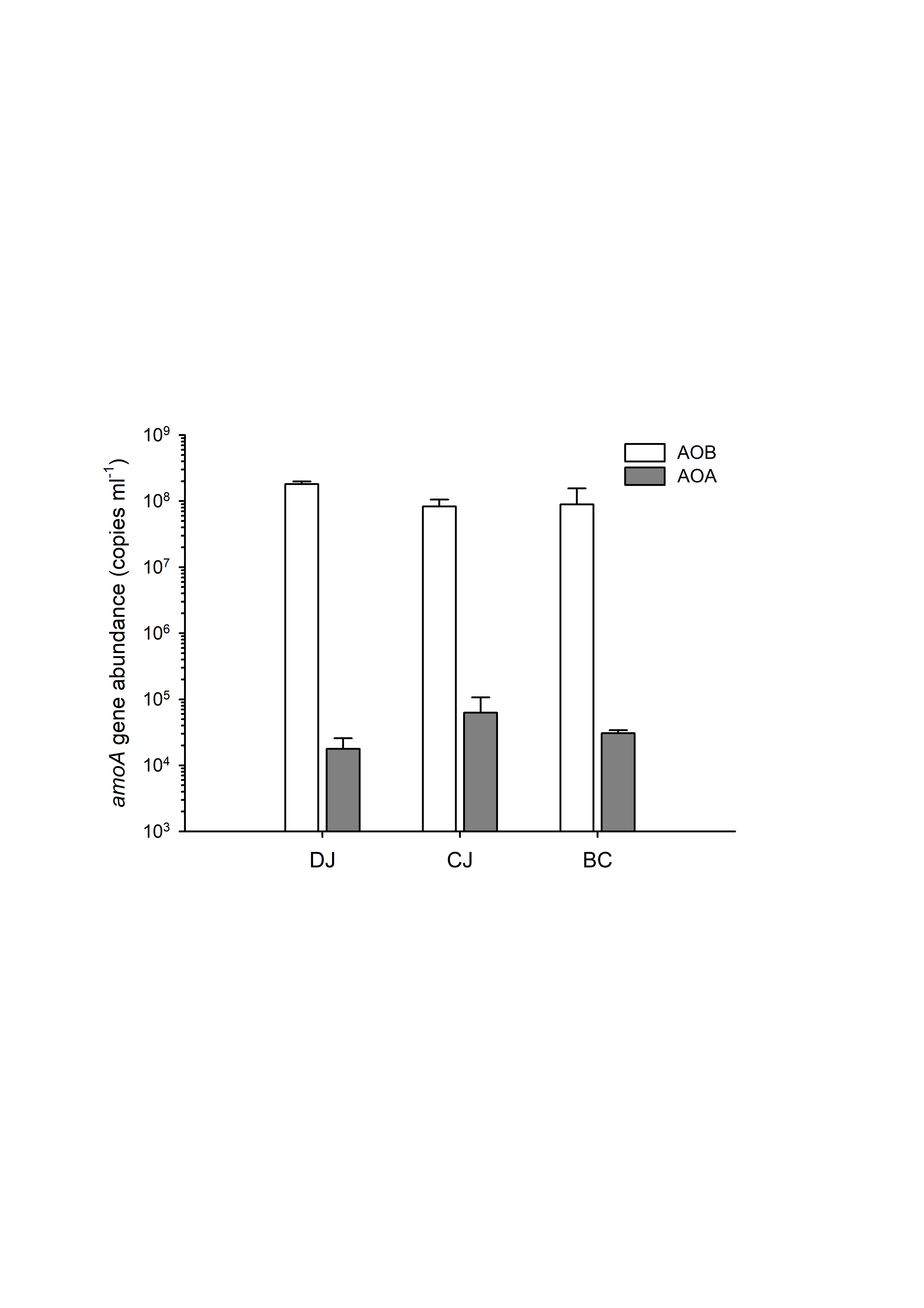


**Fig. S2.** **Inhibitory effects of various organic compounds to growth of AOA and AOB.** Four AOA strains (*N. koreense*, “*Ca.* N. chungbukensis”, “*Ca.* N. oleophilus”, and *N. viennensis*) and one AOB strain (*N. europaea*) were incubated in the AFM amended with various organic compounds. The AFM without organic compounds was used as a control. Final concentrations of organic compounds were 0.5 mM for single organic compounds and 50 mg L^-1^ for complex mixtures of organic compounds. Arginine, glutamate, lysine, valine, acetate, gluconate and salicylate amended media showed no significant inhibitory effect on the growth of all strains tested (data not shown). Error bars represent mean standard deviation for n ≥ 3 biological replicates. Significant differences between different organic compounds in each strain are indicated by different letters (One-way ANOVA, Tukey’s test, *p* < 0.001).


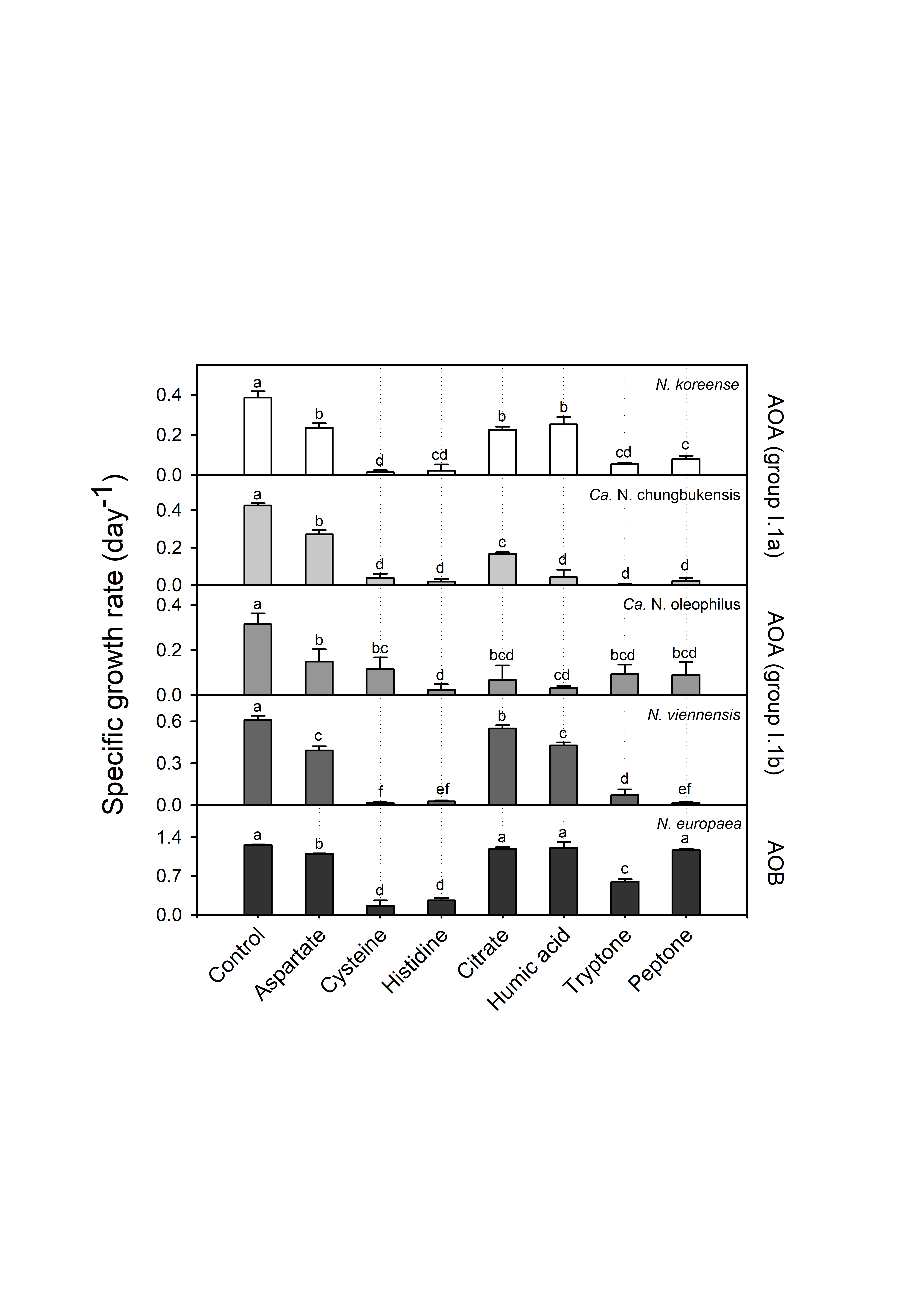


**Fig. S3. Restoration of growth of *N. viennensis* inhibited by organic compounds via augmentation of the AFM with TMS.** Growth of *N. viennensis* was fully inhibited in the presence of 0.5 mM histidine (a) or 50 mg L^-1^ complex mixture of organic compounds (b) in the AFM. Increasing the concentration of the TMS in the AFM restored growth. As a control *N. viennensis* was inoculated in the AFM with the different TMS concentrations but without supplementation of organic compounds. Error bars represent mean standard deviation for n ≥ 3 biological replicates. Significant differences between treatments are marked by different letters (Two-way ANOVA, Tukey’s test, *p* < 0.001).


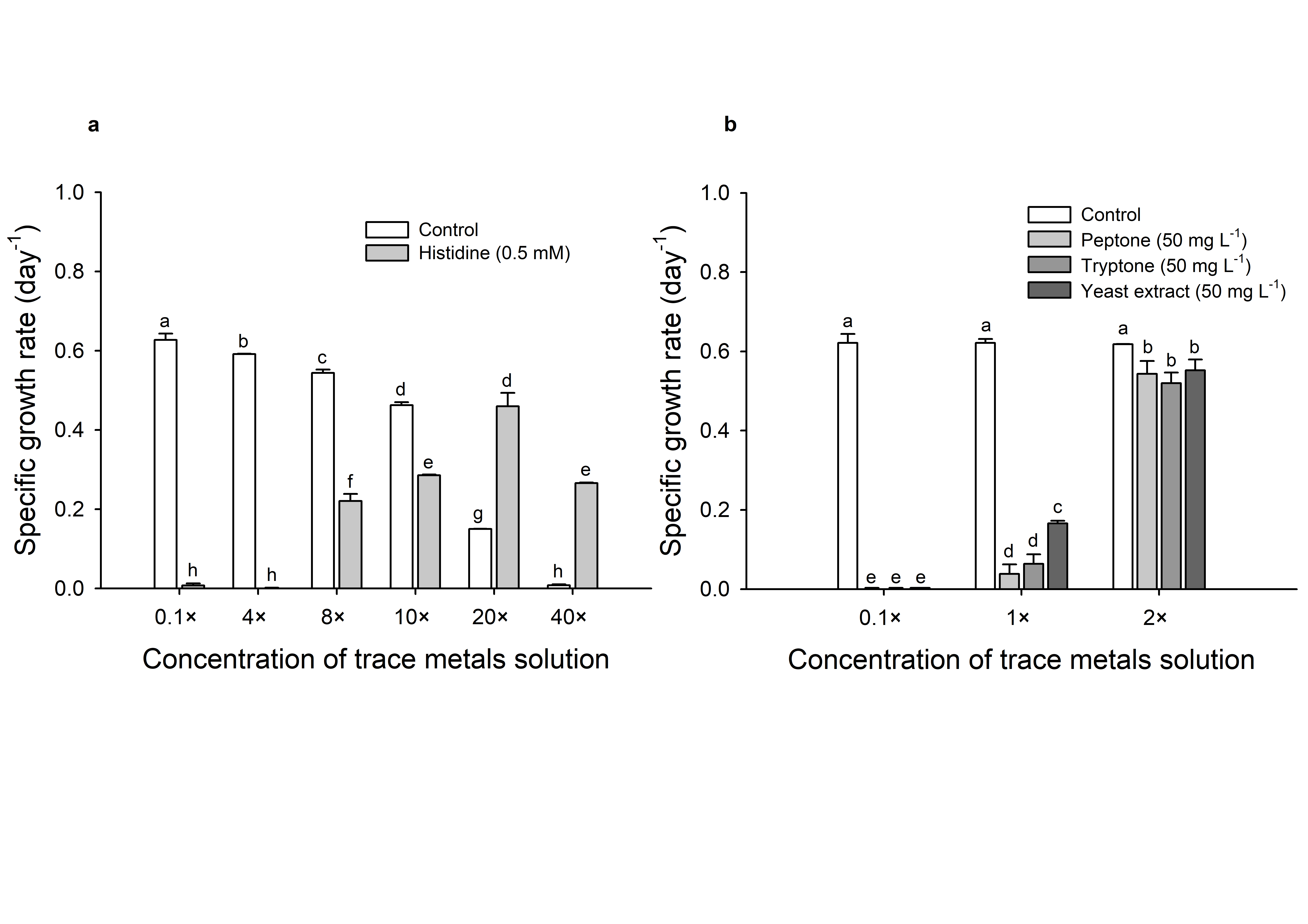


**Fig. S4. Restoration of growth of “*Ca.* N. chungbukensis” and *N. viennensis* inhibited by complex mixture of organic compounds via copper augmentation.** “*Ca.* N. chungbukensis” (a) and *N. viennensis* (b) were inoculated into the AFM supplemented with complex mixtures of organic compounds and different concentrations of copper. As a control AOA was inoculated in the AFM with the different copper concentrations but without supplementation of organic compounds. Copper concentration is indicated by times of the concentration in the TMS. Error bars represent mean standard deviation for n ≥ 3 biological replicates. Significant differences between treatments are marked by different letters (Two-way ANOVA, Tukey’s test, *p* < 0.001).


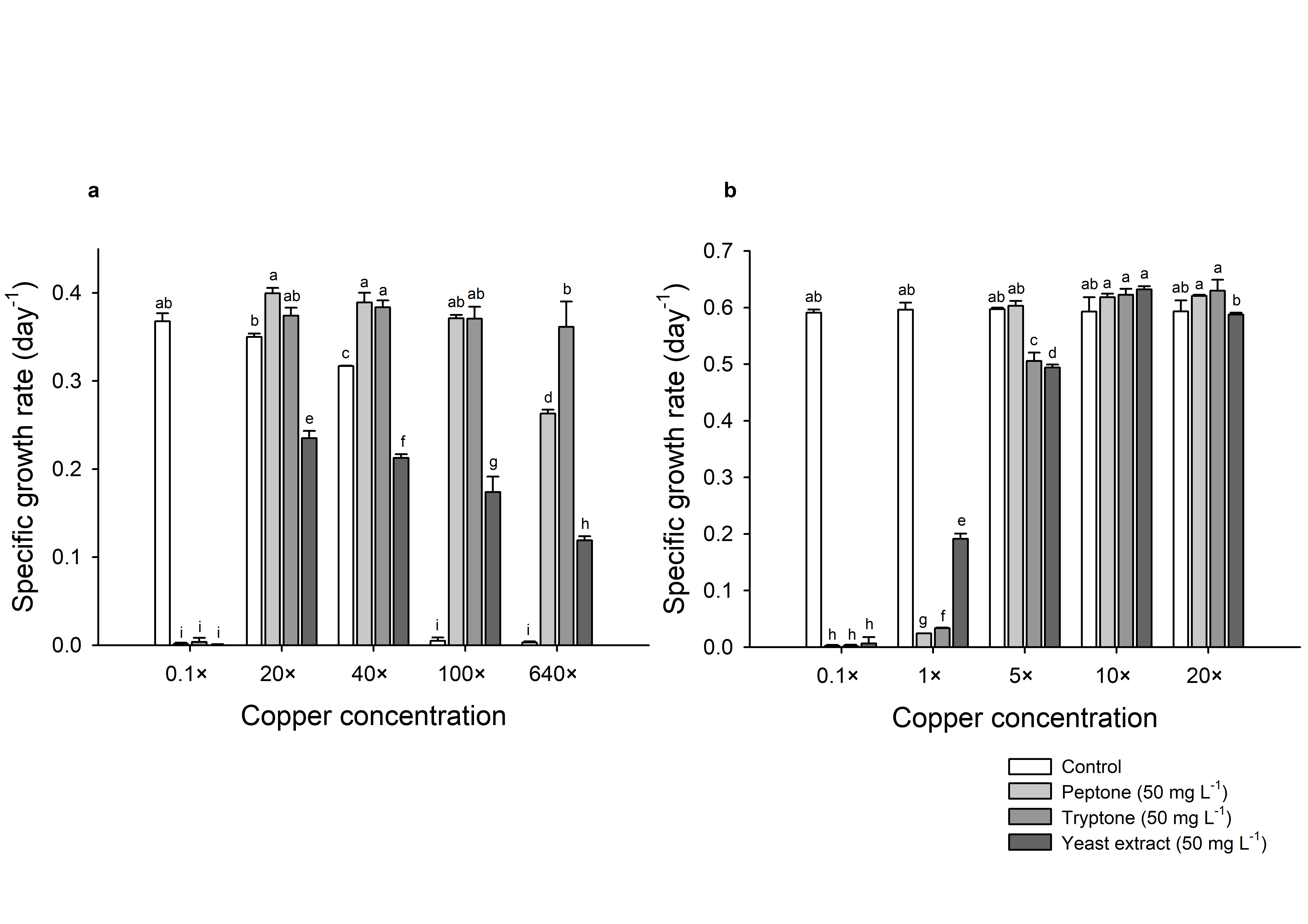


**Fig. S5. Effect of copper augmentation on growth rates of AOA and AOB in filtered wastewater.** Cells were washed by centrifugation and inoculated into filtered wastewater from the CJ plant. Various concentrations of copper were added to the wastewater. The AFM was used as a control. Copper augmentations are indicated as times of the concentration in the TMS. Error bars represent mean standard deviation for n ≥ 3 biological replicates. Significant differences between treatments in each strain are indicated by different letters (One-way ANOVA, Tukey’s test, *p* < 0.001).


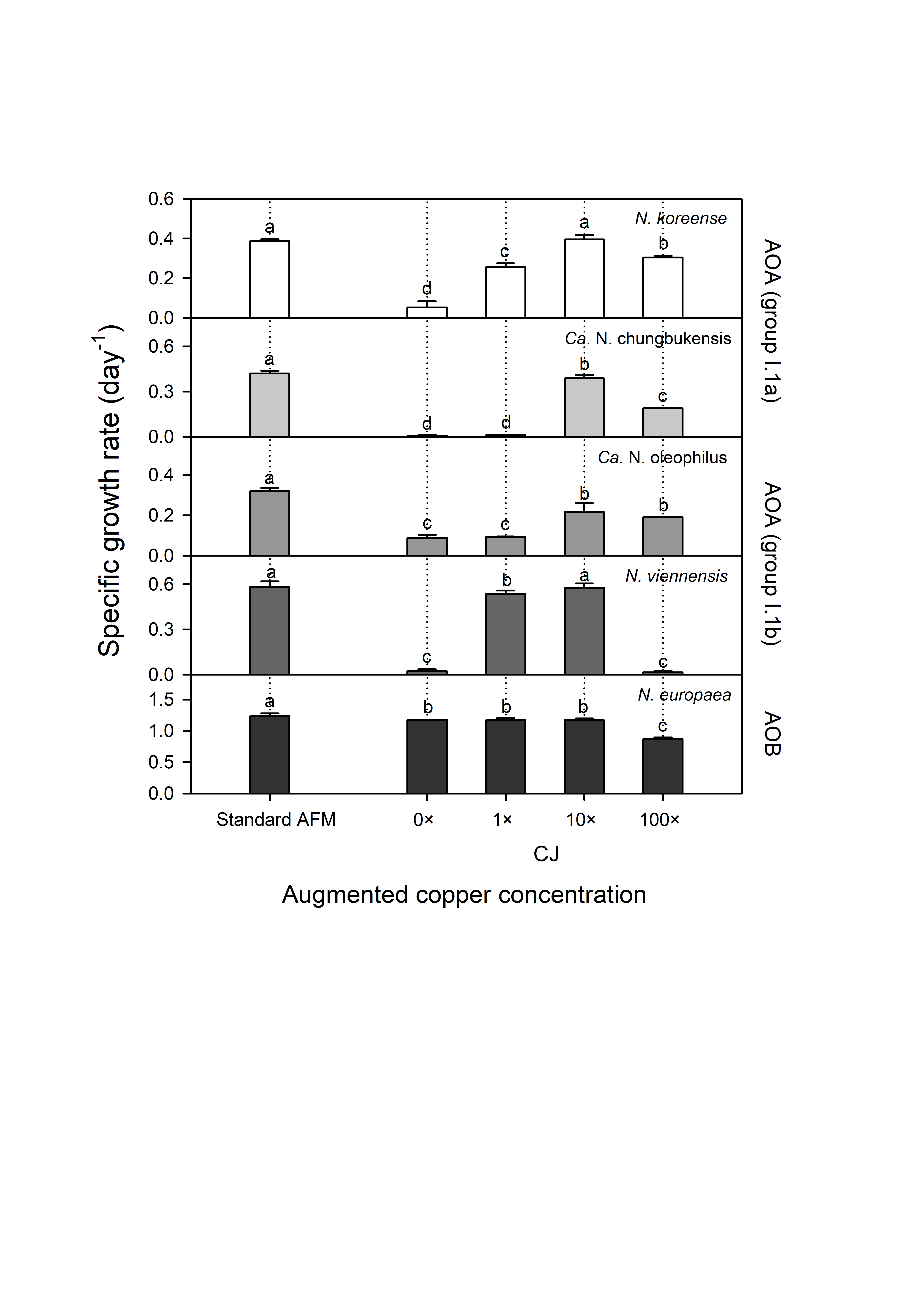


**Fig. S6. Effect of UV treatment of filtered wastewater on growth of *N. viennensis*.** Cells of *N. viennensis* were inoculated into UV-treated filtered wastewater from the plant CJ in which copper was augmented at 0×, 0.3× and 10× concentrations of that in the TMS. Filtered wastewater without UV treatment was used as a control. Error bars represent mean standard deviation for n ≥ 3 biological replicates, and significant differences between treatments are indicated by different letters (Two-way ANOVA, Tukey’s test, *p* < 0.001).


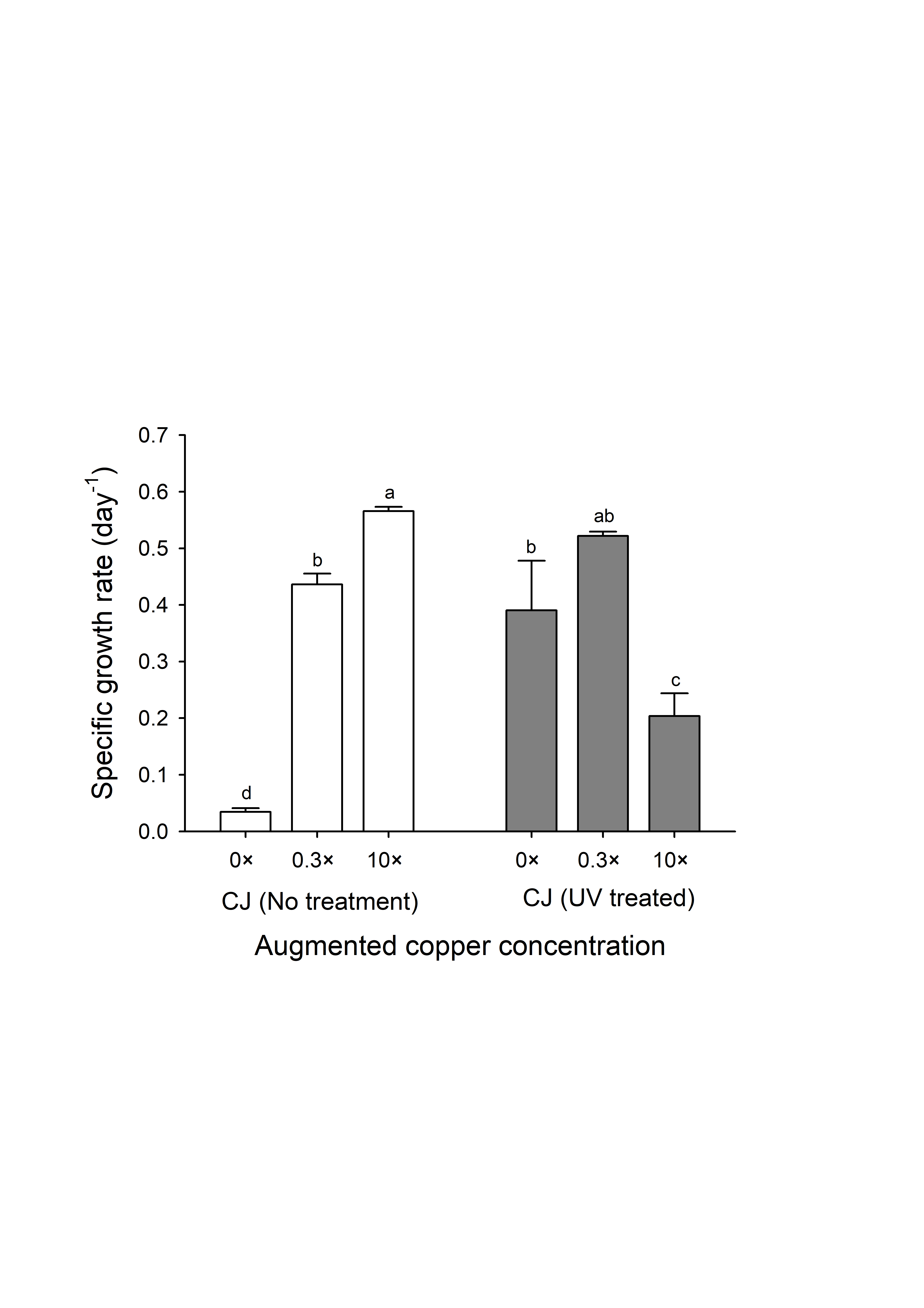


**Fig. S7. Growth rates of *N. viennensis* in filtered wastewater from WWTPs with high AOA abundances.** Wastewater from these WWTPs (Refinery D, RBC-1 and RBC-8) (Table 1) were filter-sterilized and inoculated with washed cells of *N. viennensis*. Inoculations in AFM with copper-free 0.1× TMS and filter-sterilized wastewater of the municipal, AOB- dominated WWTP CJ were used as controls. To test for the effect of copper augmentation, the wastewater samples were augmented with 10× concentration of copper in the TMS. Error bars represent mean standard deviation for n ≥ 3 biological replicates. Significant differences between treatments are indicated by different letters (Two-way ANOVA, Tukey’s test, *p* < 0.001).


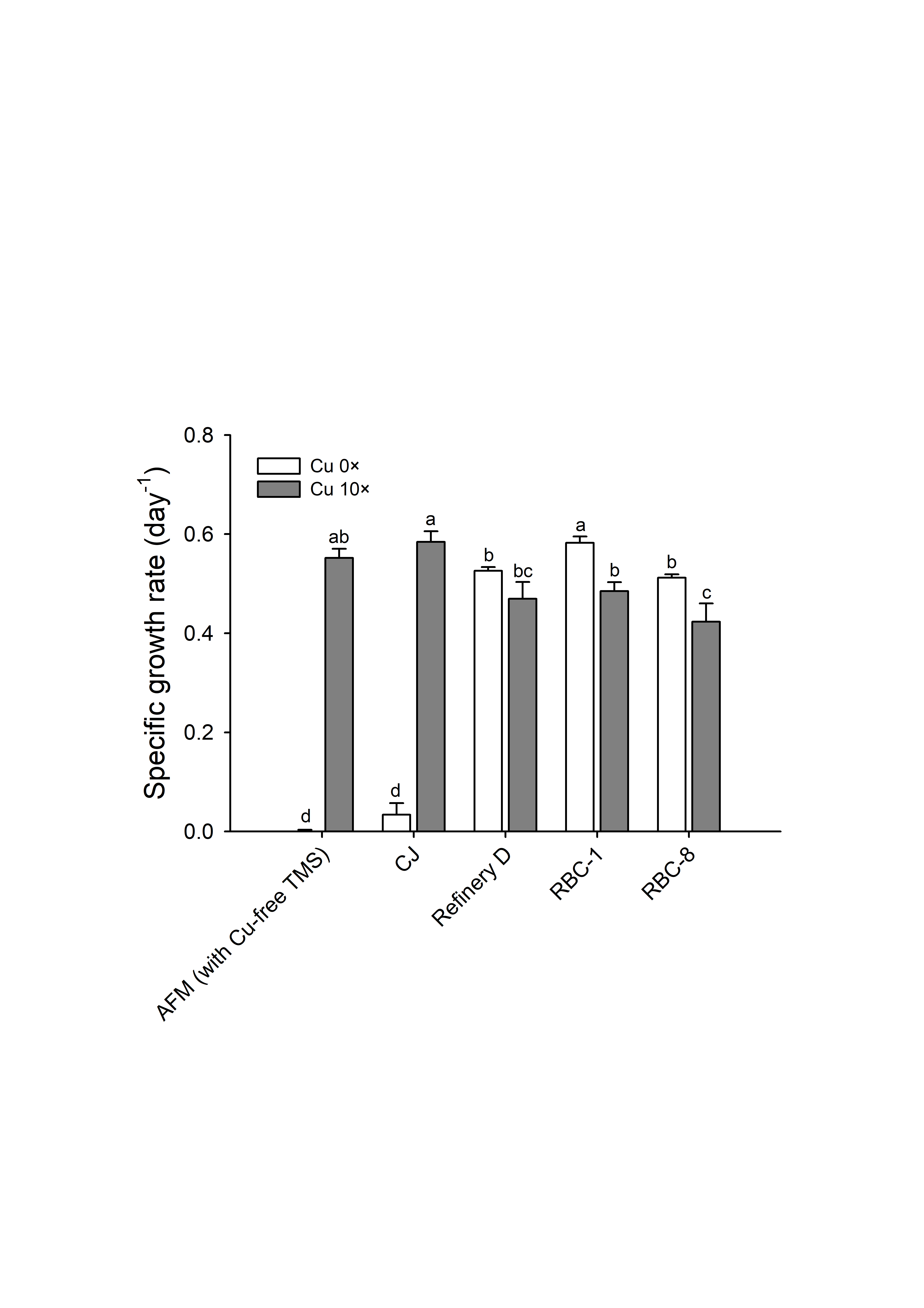


**Supplementary References**

1. Argüello JM, Raimunda D, Padilla-Benavides T. Mechanisms of copper homeostasis in bacteria. *Front Cell Infect Microbiol*. 2013;3:1-14.

2. Knapp CW, Fowle DA, Kulczycki E, Roberts JA, Graham DW. Methane monooxygenase gene expression mediated by methanobactin in the presence of mineral copper sources. *Proc Natl Acad Sci USA*. 2007;104:12040-12045.

3. Gu W, Farhan Ul Haque M, Baral BS, Turpin EA, Bandow NL, Kremmer E et al. A TonB-dependent transporter is responsible for methanobactin uptake by *Methylosinus trichosporium* OB3b. *Appl Environ Microbiol*. 2016;82:1917-1923.

4. Wang L, Zhu M. Diisonitrile natural product SF2768 functions as a chalkophore that mediates copper acquisition in *Streptomyces thioluteus*. *ACS Chem Biol*. 2017;12:3067-3075.

5. Koh EI, Henderson JP. Microbial copper-binding siderophores at the host-pathogen interface. *J Biol Chem*. 2015;290:18967-18974.

6. Jung MY, Kim JG, Sinninghe Damste JS, Rijpstra WI, Madsen EL, Kim SJ et al. A hydrophobic ammonia-oxidizing archaeon of the *Nitrosocosmicus* clade isolated from coal tar-contaminated sediment. *Environ Microbiol Rep*. 2016;8:983-992.

7. Kerou M, Offre P, Valledor L, Abby SS, Melcher M, Nagler M et al. Proteomics and comparative genomics of *Nitrososphaera viennensis* reveal the core genome and adaptations of archaeal ammonia oxidizers. *Proc Natl Acad Sci USA*. 2016;113:E7937-E7946.

8. Walker CB, de la Torre JR, Klotz MG, Urakawa H, Pinel N, Arp DJ et al. *Nitrosopumilus maritimus* genome reveals unique mechanisms for nitrification and autotrophy in globally distributed marine crenarchaea. *Proc Natl Acad Sci USA*. 2010;107:8818-8823.

9. Chain P, Lamerdin J, Larimer F, Regala W, Lao V, Land M et al. Complete genome sequence of the ammonia-oxidizing bacterium and obligate chemolithoautotroph *Nitrosomonas europaea*. *J Bacteriol*. 2003;185:2759-2773.

10. Amin SA, Moffett JW, Martens-Habbena W, Jacquot JE, Han Y, Devol A et al. Copper requirements of the ammonia-oxidizing archaeon *Nitrosopumilus maritimus* SCM1 and implications for nitrification in the marine environment. *Limnol Oceanogr*. 2013;58:2037-2045.

11. Shafiee RT, Snow JT, Zhang Q, Rickaby REM. Iron requirements and uptake strategies of the globally abundant marine ammonia-oxidising archaeon, *Nitrosopumilus maritimus* SCM1. *ISME J*. 2019;13:2295-2305.

12. Keluskar R, Nerurkar A, Desai A. Mutualism between autotrophic ammonia-oxidizing bacteria (AOB) and heterotrophs present in an ammonia-oxidizing colony. *Arch Microbiol*. 2013;195:737-747.

13. Anton A, Weltrowski A, Haney CJ, Franke S, Grass G, Rensing C et al. Characteristics of zinc transport by two bacterial cation diffusion facilitators from *Ralstonia metallidurans* CH34 and *Escherichia coli*. *J Bacteriol*. 2004;186:7499-7507.

14. Nevo Y, Nelson N. The NRAMP family of metal-ion transporters. *BBA-Mol Cell Res*. 2006;1763:609-620.

15. Pontel LB, Scampoli NL, Porwollik S, Checa SK, McClelland M, Soncini FC. Identification of a *Salmonella ancillary* copper detoxification mechanism by a comparative analysis of the genome-wide transcriptional response to copper and zinc excess. *Microbiol*. 2014;160:1659-1669.

16. Bondarczuk K, Piotrowska-Seget Z. Molecular basis of active copper resistance mechanisms in Gram-negative bacteria. *Cell Biol Toxicol*. 2013;29:397-405.

17. Wintz H, Fox T, Wu YY, Feng V, Chen W, Chang HS et al. Expression profiles of *Arabidopsis thaliana* in mineral deficiencies reveal novel transporters involved in metal homeostasis. *J Biol Chem*. 2003;278:47644-47653.

18. Argüello JM, Patel SJ, Quintana J. Bacterial Cu^+^-ATPases: models for molecular structure-function studies. *Metallomics*. 2016;8:906-914.

19. Gorman-Lewis D, Martens-Habbena W, Stahl DA. Cu(II) adsorption onto ammonia-oxidizing bacteria and archaea. *Geochim Cosmochim Acta*. 2019;255:127-143.

20. Santoro AE, Dupont CL, Richter RA, Craig MT, Carini P, McIlvin MR et al. Genomic and proteomic characterization of "*Candidatus* Nitrosopelagicus brevis": an ammonia-oxidizing archaeon from the open ocean. *Proc Natl Acad Sci USA*. 2015;112:1173-1178.

21. Jung MY, Islam MA, Gwak JH, Kim JG, Rhee SK. *Nitrosarchaeum koreense* gen. nov., sp. nov., an aerobic and mesophilic, ammonia-oxidizing archaeon member of the phylum *Thaumarchaeota* isolated from agricultural soil. *Int J Syst Evol Microbiol*. 2018;68:3084-3095.

22. Qin W, Heal KR, Ramdasi R, Kobelt JN, Martens-Habbena W, Bertagnolli AD et al. *Nitrosopumilus maritimus* gen. nov., sp. nov., *Nitrosopumilus cobalaminigenes* sp. nov., *Nitrosopumilus oxyclinae* sp. nov., and *Nitrosopumilus ureiphilus* sp. nov., four marine ammonia-oxidizing archaea of the phylum *Thaumarchaeota*. *Int J Syst Evol Microbiol*. 2017;67:5067-5079.

23. de la Torre JR, Walker CB, Ingalls AE, Konneke M, Stahl DA. Cultivation of a thermophilic ammonia oxidizing archaeon synthesizing crenarchaeol. *Environ Microbiol*. 2008;10:810-818.

24. Lehtovirta-Morley LE, Ge C, Ross J, Yao H, Nicol GW, Prosser JI. Characterisation of terrestrial acidophilic archaeal ammonia oxidisers and their inhibition and stimulation by organic compounds. *FEMS Microbiol Ecol*. 2014;89:542-552.

25. Jung MY, Kim JG, Sinninghe Damste JS, Rijpstra WI, Madsen EL, Kim SJ et al. A hydrophobic ammonia-oxidizing archaeon of the *Nitrosocosmicus* clade isolated from coal tar-contaminated sediment. Environ Microbiol Rep. 2016;8:983-992.

26. Macfarlane GT, Herbert RA. Effect of oxygen tension, salinity, temperature and organic matter concentration on the growth and nitrifying activity of an estuarine strain of *Nitrosomonas*. *FEMS Microbiol Lett*. 1984;23:107-111.

27. Clark C, Schmidt EL. Growth response of *Nitrosomonas europaea* to amino acids. *J Bacteriol*. 1967;93:1302-1308.

28. Ehrich S, Behrens D, Lebedeva E, Ludwig W, Bock E. A new obligately chemolithoautotrophic, nitrite-oxidizing bacterium,*Nitrospira moscoviensis* sp. nov. and its phylogenetic relationship. *Arch Microbiol*. 1995;164:16-23.

29. Vardanyan NS, Akopyan VP. *Leptospirillum*-Like bacteria and evaluation of their role in pyrite oxidation. *Microbiol*. 2003;72:438-442.

30. Braley SA, Sr., Kinsel NA, Leathen WW. *Ferrobacillus ferrooxidans*: a chemosynthetic autotrophic bacterium. *J Bacteriol*. 1956;72:700-704.

31. Johnson CL, Vishniac W. Growth Inhibition in *Thiobacillus neapolitanus* by Histidine, Methionine, Phenylalanine, and Threonine. *J Bacteriol*. 1970;104:1145-1150.

32. Pol A, Heijmans K, Harhangi HR, Tedesco D, Jetten MSM, Op den Camp HJM. Methanotrophy below pH 1 by a new *Verrucomicrobia* species. *Nature*. 2007;450:874.

33. Eroshin VK, Harwood JH, Pirt SJ. Influence of amino acids, carboxylic acids and sugars on the growth of *Methylococcus capsulatus* on methane. *J Appl Microbiol*. 1968;31:560-567.

34. Heinrich A. Differential sensitivity of a coccal green algal and a cyanobacterial species to dissolved natural organic matter (NOM) (8 pp). *Environ Sci Pollut Res Int*. 2007;14 Suppl 1:11-18.

35. Nagai T, Imai A, Matsushige K, Fukushima T. Effect of iron complexation with dissolved organic matter on the growth of cyanobacteria in a eutrophic lake. *Aquat Microb Ecol* 2006;44:231-239.

36. Rappé MS, Connon SA, Vergin KL, Giovannoni SJ. Cultivation of the ubiquitous SAR11 marine bacterioplankton clade. *Nature*. 2002;418:630-633.

37. Button DK, Schut F, Quang P, Martin R, Robertson BR. Viability and isolation of marine bacteria by dilution culture: theory, procedures, and initial results. *Appl Environ Microbiol*. 1993;59:881-891.

38. Tourna M, Stieglmeier M, Spang A, Konneke M, Schintlmeister A, Urich T et al. *Nitrososphaera viennensis*, an ammonia oxidizing archaeon from soil. Proc Natl Acad Sci USA. 2011;108:8420-8425.

39. Stieglmeier M, Klingl A, Alves RJ, Rittmann SK, Melcher M, Leisch N et al. *Nitrososphaera viennensis* gen. nov., sp. nov., an aerobic and mesophilic, ammonia-oxidizing archaeon from soil and a member of the archaeal phylum *Thaumarchaeota*. *Int J Syst Evol Microbiol*. 2014;64:2738-2752.

40. Francis CA, Roberts KJ, Beman JM, Santoro AE, Oakley BB. Ubiquity and diversity of ammonia-oxidizing archaea in water columns and sediments of the ocean. Proc Natl Acad Sci USA. 2005;102:14683-14688.

41. Rotthauwe JH, Witzel KP, Liesack W. The ammonia monooxygenase structural gene amoA as a functional marker: molecular fine-scale analysis of natural ammonia-oxidizing populations. *Appl Environ Microbiol*. 1997;63:4704-4712.

42. Avrahami S, Liesack W, Conrad R. Effects of temperature and fertilizer on activity and community structure of soil ammonia oxidizers. *Environ Microbiol*. 2003;5:691-705.

43. Chain P, Lamerdin J, Larimer F, Regala W, Lao V, Land M et al. Complete genome sequence of the ammonia-oxidizing bacterium and obligate chemolithoautotroph *Nitrosomonas europaea*. J Bacteriol. 2003;185:2759-2773.

44. Anton A, Weltrowski A, Haney CJ, Franke S, Grass G, Rensing C et al. Characteristics of zinc transport by two bacterial cation diffusion facilitators from *Ralstonia metallidurans* CH34 and *Escherichia coli*. J Bacteriol. 2004;186:7499-7507.

45. Pontel LB, Scampoli NL, Porwollik S, Checa SK, McClelland M, Soncini FC. Identification of a *Salmonella ancillary* copper detoxification mechanism by a comparative analysis of the genome-wide transcriptional response to copper and zinc excess. Microbiology (Reading, Engl). 2014;160:1659-1669.

46. Bondarczuk K, Piotrowska-Seget Z. Molecular basis of active copper resistance mechanisms in Gram-negative bacteria. Cell Biol Toxicol. 2013;29:397-405.

47. Wintz H, Fox T, Wu YY, Feng V, Chen W, Chang HS et al. Expression profiles of *Arabidopsis thaliana* in mineral deficiencies reveal novel transporters involved in metal homeostasis. J Biol Chem. 2003;278:47644-47653.

48. Daims H, Lebedeva EV, Pjevac P, Han P, Herbold C, Albertsen M et al. Complete nitrification by *Nitrospira* bacteria. Nature. 2015;528:504-509.
